# Land sharing complements land sparing in the conservation of disturbance-dependent species

**DOI:** 10.1007/s13280-022-01820-1

**Published:** 2022-12-24

**Authors:** Malin Tälle, Erik Öckinger, Therese Löfroth, Lars B. Pettersson, Henrik G. Smith, Martin Stjernman, Thomas Ranius

**Affiliations:** 1 Department of Ecology, Swedish University of Agricultural Sciences, Box 7044, 750 07 Uppsala, Sweden; 2 Department of Wildlife, Fish and Environmental Studies, Swedish University of Agricultural Sciences, 901 83 Umeå, Sweden; 3 Department of Biology, Biodiversity Unit, Lund University, Ecology Building, 223 62 Lund, Sweden; 4 Centre for Environmental and Climate Science, Lund University, Ecology Building, 223 62 Lund, Sweden

**Keywords:** Fire, Forest, Grazing, Mowing, Protected area, Semi-natural grassland

## Abstract

Alteration of natural disturbances in human-modified landscapes has resulted in many disturbance-dependent species becoming rare. Conservation of such species requires efforts to maintain or recreate disturbance regimes. We compared benefits of confining efforts to habitats in protected areas (a form of land sparing) versus integrating them with general management of production land (a form of land sharing), using two examples: fire in forests and grazing in semi-natural grasslands. We reviewed empirical studies from the temperate northern hemisphere assessing effects of disturbances in protected and non-protected areas, and compiled information from organisations governing and implementing disturbances in Sweden. We found advantages with protection of areas related to temporal continuity and quality of disturbances, but the spatial extent of disturbances is higher on production land. This suggests that an approach where land sparing is complemented with land sharing will be most effective for preservation of disturbance-dependent species in forests and semi-natural grasslands.

**Supplementary Information:**

The online version contains supplementary material available at 10.1007/s13280-022-01820-1.

## Introduction

Natural ecosystems are often strongly influenced by large-scale disturbances, which by creating or maintaining early-successional habitats generate temporal dynamics and spatial heterogeneity. In terrestrial habitats, disturbances often decrease the cover of trees and other vegetation. Species communities are adapted to natural disturbance regimes, and biodiversity can be favoured by disturbances suppressing dominating species, thereby reducing the risk of competitive exclusions (White and Jentsch 2001; Walker 2011). Species adapted to disturbances can occur in early-successional stages, immediately after disturbances have taken place, while others occur in habitats at a later stage. Species adapted to early stages are typically characterised by good dispersal abilities and high reproductive output, but unless the disturbance occurs frequently, they are soon out-competed by species that are stronger competitors but slower to colonise after a disturbance (Grime 1977). Hence, the magnitude and frequency of disturbances are important drivers of community composition (Vellend 2016). Among species inhabiting early-successional habitats, there is a gradient from highly specialised species dependent on certain characteristics of the disturbances, e.g. burned wood or grazing (Goodman and McCravy 2008; Bråthen et al. 2021), to generalist species preferring early-successional habitats in general, e.g. sun-exposed habitats (Rubene et al. 2014).

As a result of changes in disturbance regimes caused by human activities (Cremene et al. 2005; Navarro et al. 2015; Kelly et al. 2020), early-successional species adapted to disturbances like fire, grazing by large herbivores, or flooding are becoming increasingly rare (Brawn et al. 2001; Lawler et al. 2002; Hanberry 2014). Traditionally, nature conservation has focussed on preserving species adapted to late-successional habitats (Brotons et al. 2003; Bouget et al. 2014), but conservation of species dependent on disturbances requires maintenance or restoration of disturbance regimes, either by promoting the occurrence of natural disturbances, or by replacing these with human-induced ones. This can be accomplished either through management actions in protected areas or through conservation efforts that are integrated within production systems. We argue that these two options can be conceptualised as a special case of land sparing and land sharing. Land sparing means that biodiversity conservation efforts and production are spatially separated, such that conservation efforts are concentrated to land set aside for this purpose, while the other land is used for production. In contrast, land sharing means that production and biodiversity conservation are spatially integrated, such that forestry and agricultural management are adapted to also promote biodiversity (Green et al. 2005; Edwards et al. 2014; Sidemo-Holm et al. 2021). An unexplored question is whether land sparing or sharing benefits disturbance-dependent species the most. Protection of land can be an effective way of preventing loss of habitat and biodiversity (Leverington et al. 2010; Watson et al. 2014), and result in high nature values. However, protected areas are few, and may be too small for natural disturbance dynamics to operate adequately (Baker 1992; Brackhane et al. 2021). In contrast, production landscapes typically contain a large proportion of early-successional habitats, and management practices that might emulate natural disturbances (Rubene et al. 2014; Batáry et al. 2015; Dániel-Ferreira et al. 2020).

Here, we evaluate this question using two disturbances as examples: fire in forests and grazing of semi-natural grasslands. These disturbances have historically impacted large parts of boreal and temperate biomes of the northern hemisphere, with many species dependent on them (Wikars 2002; Wilson et al. 2012; Kayes and Mallik 2020). They represent two endpoints in the spectrum of disturbances regarding frequency and magnitude. Forest fires occur relatively rarely but completely transform the habitat where they occur. Since natural forest fires are unpredictable in space and time and burned forests are only used by specialised species during a limited time, most species confined to burned forests have a high capacity for long-distance dispersal or can remain in the seed bank for decades (Kouki et al. 2012; Risberg and Granström 2012; Heikkala et al. 2017). In contrast, grassland species, especially plants, often have a more limited dispersal range (Bischoff 2002; Maurer et al. 2003) and depend on disturbance at smaller spatio-temporal scales.

Forest fires have shaped post-glacial boreal and hemiboreal forests, and temperate coniferous forests (Zackrisson 1977; Bengtsson et al. 2000; Niklasson et al. 2010; Adámek et al. 2015), and are vital for species that either require high temperatures for reproduction, or utilise burned soil or wood (pyrophilous species) (Granström and Schimmel 1993; Wikars 2002; Olsson and Jonsson 2010). Fires also favour species dependent on dead wood (saproxylic species), especially those associated with sun-exposed conditions (Toivanen and Kotiaho 2007; Ylisirniö et al. 2012). In the long term, fires and other disturbances also affect tree species composition and openness of the forest landscapes (Mekonnen et al. 2019). Fires have deliberately been suppressed to benefit production forestry (Wallenius 2011). For example, between 1500 and 1850, average fire intervals in Fennoscandia were about 80 years, but they are now in the hundreds of thousands of years (Zackrisson 1977; Granström and Niklasson 2008; Wallenius 2011). This is negatively affecting species dependent on fire (Ryan et al. 2013; Eales et al. 2018). For that reason, prescribed burning (i.e. strictly controlled burning of smaller areas) is a frequent conservation measure (Toivanen and Kotiaho 2007; Vanha-Majamaa et al. 2007; Olsson and Jonsson 2010). In northern Europe, prescribed burning is performed as part of management of protected forests, or as part of forest certification in managed forest by Forest Stewardship Council (FSC) or the Programme for the Endorsement of Forest Certification (PEFC).

Semi-natural grasslands are shaped by disturbances from traditional agricultural management, such as grazing and mowing. These reduce aboveground-biomass and prevent encroachment of woody species, thereby keeping grasslands open and maintaining nutrient-poor conditions (Hautier et al. 2009; Oelmann et al. 2009). Such conditions are essential for plant species that are weak competitors for light and nutrients, as well as for many insects, birds, and other species (Perkins et al. 2000; Öckinger and Smith 2006; Habel et al. 2013). Grassland habitats were originally formed by grazing by large herbivores which were abundant during the last two million years before they were hunted to extinction, or became domesticated (Bråthen et al. 2021). During the latest few thousand years in Europe, traditional agricultural systems with management in the form of grazing and mowing have played a vital part in maintaining grasslands (Poschlod and WallisDeVries 2002; Hejcman et al. 2013; Eriksson 2020). The strong decrease in semi-natural grasslands following intensification of agriculture (Firbank 2005; Cousins et al. 2015; Ridding et al. 2015) has resulted in some grasslands being protected to maintain management and preserve biodiversity. Within the EU, agri-environmental schemes (AES) have also included measures aimed at preserving semi-natural grasslands and associated species, through maintenance of traditional management (Batáry et al. 2015; Jordbruksverket 2021).

Whether conservation efforts targeting disturbance-dependent species in forests and semi-natural grasslands should mainly be performed in protected areas or integrated in production landscapes is a key question for future conservation strategies. To answer this, we need both empirical knowledge about how species are affected by disturbance in a protected and non-protected context, and the societal context, especially the degree to which existing policy and governance affect the possibility of implementing these strategies (cf. Kremen 2015). For this purpose, we reviewed (i) the scientific literature comparing effects of fires and grazing or mowing on disturbance-dependent species in protected and non-protected forests and semi-natural grasslands; and (ii) information from policy-makers and practitioners on the temporal and spatial continuity of disturbances and characteristics of disturbances related with method, intensity, and post-disturbance treatment, using Sweden as a case study. In Sweden, forest fires and grazing and mowing of grasslands are the two most important disturbances considered in nature conservation. While evaluations of land sparing and sharing strategies typically assess biodiversity benefits given a target to maintain high production (Sidemo-Holm et al. 2021), or maybe more relevant in this context, against a target of equal costs for society to implement the alternative strategies, we did not consider these aspects.

## Methods

### Description of study systems

In 2017, forests covered 28 million ha of Sweden, with almost 9% being protected with a bias towards low-productive and northerly areas (Riksskogstaxeringen 2018; Statistiska Centralbyrån 2020). While there are no official statistics on the area of protected and non-protected forests burned each year, a study by Ramberg et al. (2018) concluded that about 0.006% of the total forest area burned annually during 2011–2015. Of the 460 000 ha of semi-natural grasslands in Sweden, about 5% were protected in 2017 (Christensen et al. 2015; Statistiska Centralbyrån 2018). The area of remaining semi-natural grasslands is just a small fraction of the area 100 years ago. For example, Cousins et al. (2015) estimated that 96% of the semi-natural grassland area had been lost since year 1900 in one region in Sweden.

Sweden has adopted both land sparing and land sharing strategies for the preservation of disturbance-dependent species. Land sparing strategies consist of prescribed burning or grassland management in government-based or voluntary assigned protected areas. Land sharing strategies consist either of prescribed burning of non-protected forests to fulfil the requirements of FSC- or PEFC-certified forests or with the help of government subsidies (Forest Stewardship Council 2020; Skogsstyrelsen 2021b), or extensive management of non-protected semi-natural grasslands incentivised by AES payments (Knop et al. 2006; Beaufoy et al. 2011; Science for Environment Policy 2017).

### Literature review of effects on disturbance-dependent species

To summarise the scientific knowledge about the effects of disturbances on disturbance-dependent species in protected vs. non-protected areas, we systematically reviewed the available scientific literature. We focussed on studies explicitly comparing consequences of forest fires and grazing or mowing of semi-natural grasslands in protected and non-protected areas.

#### Search strategy

We searched for peer-reviewed studies in academic publications in April 2021, using the bibliographic database Scopus. The search fields title, abstract, and keywords were used, with search terms limited to the English language. We performed internet searches for grey literature, i.e. evidence not published in peer-reviewed publications, in March 2021, using Google Search (google.com) and Google Scholar (scholar.google.com) (in incognito mode). We used search terms in English in Google, and search terms in Swedish in both Google and Google Scholar. Multiple separate search strings were used for these searches. As recommended by Haddaway et al. (2015), we only included the first 200 search results, but the number of results rarely exceeded 200. We performed separate searches, using separate search strings for studies focussing on effects of fire in forests and grazing or mowing in semi-natural grasslands. All search terms used are detailed in Table S1.

#### Screening process

We compiled all articles found and removed duplicates. After this, we screened articles using an established set of eligibility criteria (see  “Eligibility criteria” section), with separate screening of scientific and grey literature. Articles were first screened based on title, followed by abstract, and lastly full text. If there was any doubt about the relevance of an article during title or abstract screening, we retained it for the next stage of assessment. During the screening process, we noted the reason for exclusion of each article. The number of records returned from the searches and the number of records retained and excluded during each step of the screening process are detailed in Fig. S1. After full-text screening, we compiled information about all included studies in a database, along with relevant information for each study, as well as the main conclusion(s). We did not perform any meta-analyses, instead basing conclusion on the results of individual studies on reported statistical tests or in their absence, other presentation of the results.

#### Eligibility criteria

We used the following eligibility criteria for inclusion, both for peer-reviewed and grey literature. Studies had to be performed either in forests where fire (either natural forest fire or prescribed burning) had taken place, or in semi-natural grasslands that were grazed or mowed. In addition, effects had to be compared between formally protected areas and non-protected areas. Effects had to be examined on disturbance-dependent species, or important structures resulting from disturbances. In forests, these are pyrophilous or saproxylic species, while important structures include burned and dead wood occurring after fire. In semi-natural grasslands, disturbance-dependent species are those benefitting from the open conditions created by large grazers, e.g. grassland plant species, and animal and fungal species that either depend directly on these plant species or on the vegetation structure in grasslands, e.g. pollinators and specialised herbivores. For both fire and grazing, we included studies examining at least one of the following response variables: diversity of disturbance-dependent species (e.g. species richness and diversity, and genetic, phylogenetic, or functional diversity); composition, occurrence, and abundance of disturbance-dependent species; reproductive and colonisation success; and persistence and extinction risk of disturbance-dependent species or populations. We included studies assessing effects at a single or multiple time points. Biodiversity and natural disturbance regimes vary widely among biomes, which may generate systematic differences. Therefore, we only included studies performed in the temperate climatic zone of the Northern hemisphere. We included any study performed only in part within this zone if the majority of the studied sites was within the geographic zone, or if it was possible to separate conclusions based on the geography. All included countries are detailed in Table S2.

### Information about governance and implementation of disturbances

We obtained information on how disturbances are governed and implemented from governmental agencies, companies, and other organisations responsible for maintaining disturbances in forests and semi-natural grasslands in Sweden. We were particularly interested in differences between land sparing and land sharing in (i) the temporal continuity of disturbances related to planning and funding of disturbances, (ii) monitoring of effects of disturbances, (iii) the spatial continuity of disturbances, and (iv) the characteristics of disturbances such as method, timing, intensity, and post-disturbance treatment. We performed dedicated searches, using Swedish search terms within specific homepages or using Google Search, to find information from Swedish governmental agencies, companies, and other organisations. This included the Swedish Environmental Protection Agency (Swedish EPA, responsible for proposing and implementing environmental policies), the Swedish Forestry Agency (SFA, the national authority in charge of forest-related issues), the Swedish Board of Agriculture (SBA, the national authority in the field of agricultural and food policy, and responsible for AES payments), County Administration Boards (CABs, responsible for e.g. management of nature reserves and administrating AES controls and payments), forest companies, and FSC Sweden. Some searches were systematic, such as searching homepages using search terms similar to those used for the review of empirical evidence (Table S1). Others were performed using Google Search to answer specific questions. In addition, we e-mailed 35 persons whose work related to disturbances in forests and semi-natural grasslands and protection of areas at the organisations and asked questions (Appendix S1). We contacted four CABs, in southern (Skåne), south-central (Uppsala), and northern (Västernorrland and Västerbotten) Sweden, asking similar questions. We also contacted four large forest companies operating in Sweden. Of the 24 persons responding, most replied to queries through e-mail, but in some cases, we performed interviews through video conference calls. At least one and often a few persons from each organisation contacted provided responses. To ensure anonymity, we present the information provided by the respondents in a general form, rather than as attributed to specific respondents.

We examined 65 randomly selected management plans from Skåne, Uppsala, and Västernorrland counties for information on how disturbances are considered. These were available through the Swedish EPA’s database of protected areas (Naturvårdsverket 2020).

## Results

### Literature review of effects on disturbance-dependent species

In total, the full text of 91 studies related to forests and 553 studies related to semi-natural grasslands were assessed against the eligibility criteria, resulting in the inclusion of five and 21 studies, respectively (Table 1, Appendix S2).

**Table 1:** Details on included studies comparing effects of disturbances in protected and non-protected forests (five studies) and semi-natural grasslands (21 studies). Numbers in parentheses are the number of studies for each category. Note that a single study sometimes investigated more than one organism and response, and occasionally were performed in more than one country. Snapshot studies compared effects at a single time point

Organisms studied	
Forest	Saproxylic insects (3), Dead wood (2), Pyrophilous insects (1)
Semi-natural grassland	Plants (11), Butterflies (5), Birds (2), Carabid beetles (2), Grasshoppers (2), Arthropods (1)
Response	
Forest	Abundance (3), Species richness (3), Volume dead wood (2), Species composition (1), Species occurrence (1)
Semi-natural grassland	Species richness (17), Abundance (6), Species composition (6), Shannon diversity (2), Beta diversity (1), Egg density (1), Floristic value (1), Genetic diversity (1), Jaccard similarity (1), Shannon evenness (1), Species cover (1), Species density (1), Species occurrence (1)
Geography	
Forest	Finland (3), Sweden (3)
Semi-natural grassland	Germany (6), The Netherlands (4), France (2), Sweden (2), USA (2), Canada (1), Czech Republic (1), England (1), Ireland (1), Poland (1), Switzerland (1), Ukraine (1)
Study length	
Forest	Snapshot (1), 1–5 years (4)
Semi-natural grassland	Snapshot (10), 1–9 years (4), 10–50 years (7)
Comparison of protection status?	
Forest	No (5)
Semi-natural grassland	Yes (12), No (9)

**Fig. 1:**
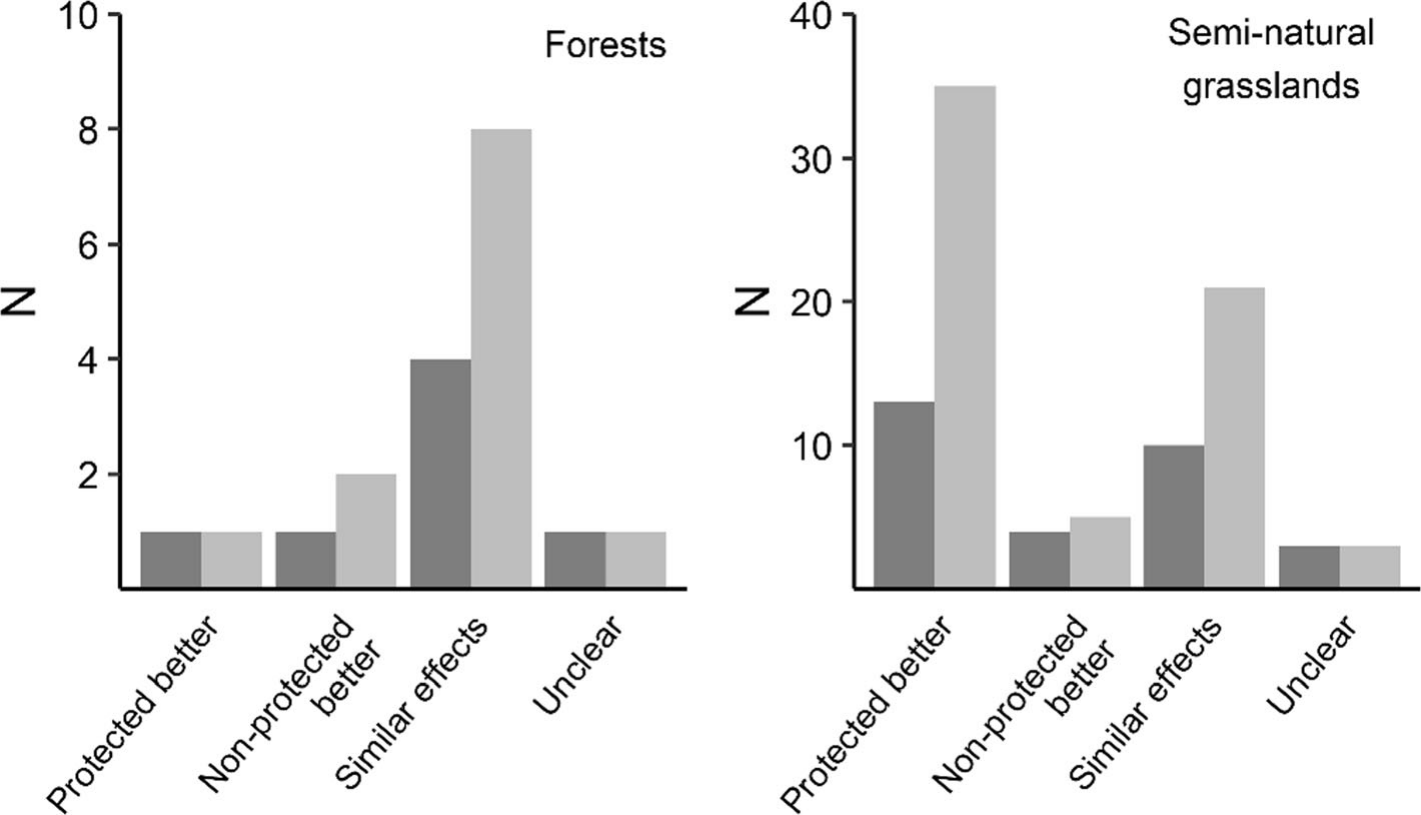
The number of studies (dark grey) or tests (light grey) that found either higher abundance or diversity of disturbance-dependent species in protected areas (“Protected better”) or non-protected areas (“Non-protected better”), similar abundance or species diversity in protected and non-protected areas, or unclear effects

The five forest studies performed 12 comparisons of the effects of fire in protected and non-protected forests. In most studies, the aim was not to compare the effect of protection status on the response to fire. Thus, no significance tests on the difference of effects between specifically protected and non-protected forests were performed. Eight of the comparisons revealed similar richness or abundance of fire-dependent species regardless of protection status. For the four remaining comparisons, the results pointed in different directions (Fig. 1). All of the included studies were short term, following the response to fire for 0–5 years, and were performed in Sweden or Finland. The studies mainly examined effects on saproxylic species, or the volume of dead wood. The only comparison focussing on pyrophilous species found similar species richness of pyrophilous beetles in protected and non-protected forests (Bohman 2009). Three of the five included studies examined effects of burning per se in standing forests, while two studies compared effects of burning in protected, standing forests, and production forests cut before burning, with varying levels of tree retention in sites (0, 10, or 50 m3 standing trees per ha) (Hyvärinen et al. 2005, 2006).

The 21 grassland studies performed 64 comparisons of protected and non-protected areas managed by grazing or mowing. In most comparisons (55%), the diversity of disturbance-dependent species was higher in protected areas, while in one-third of comparisons diversity was similar regardless of protection status (Fig. 1). Sixty-six percent of studies were snapshot studies, i.e. comparing the response using one time point, or examined short-term responses of e.g. grassland restoration across 1–9 years and the rest of the response across 10–50 years (median 20 years). Two of the studies were performed in North America, and the rest in Europe, with over one-third of studies performed in Germany and the Netherlands. Most studies examined effects on the species richness or abundance of grassland plants (37%) or insects (40%). Higher diversity in protected areas were mainly found for grassland plants, while diversity for protected and non-protected areas were similar for grassland insects. In many studies, the aim was not to compare the effect of protection status on how biodiversity respond to disturbance. For example, almost two-thirds of studies used protected grasslands as reference sites and compared them to restored grasslands located on former agricultural land. In many cases, while the species richness and abundance of grassland species increased over time in these sites, it was in many cases still lower compared to protected grasslands (e.g. Coiffait-Gombault et al. 2012; Hofmann et al. 2020). Of the studies that did compare protected and non-protected grasslands under more similar conditions, a majority still revealed a higher diversity of disturbance-dependent species in protected areas. Three of these studies examined diversity in comparable grasslands over long time periods, and found that over 30–50 years, the species richness of plants generally increased in protected areas, while it decreased in areas without formal protection (de Snoo et al. 2012; Wesche et al. 2012; Krause et al. 2015). Another long-term study found similar abundance of a grassland butterfly species in protected and non-protected areas across 10 years (Brereton et al. 2008).

### Information from organisations about governance and implementation of disturbances

#### Continuity of disturbances

##### Temporal continuity of disturbances

Of the 45 examined management plans for protected forests, few from southern and central Sweden but most from northern Sweden included prescribed burning as part of the management. Half of those prescribing fire specified that fire should occur more than once or at regular intervals. For certified managed forest, the requirement of burning 5% of the forest regeneration area over five years contributes to fire continuity. Furthermore, FSC and PEFC standards specify that it is preferable with burning of areas previously affected by fire (Programme for the Endorsement of Forest Certification 2017; Forest Stewardship Council 2020).

All of the 20 examined management plans for protected semi-natural grassland specified that annual grazing or mowing should occur indefinitely. Of these, some only specified that grazing or mowing should occur, while others detailed the method, intensity, and frequency. To some extent, non-protected semi-natural grasslands receiving AES payments are managed across long time periods as well, as farmers receiving AES payments commit to management for a five-year period (Jordbruksverket 2020), and personnel at SBA and CABs stated that a majority of them extend their management commitment beyond the initial five-year period.

Governmental funding to ensure that planned disturbances take place is available for both protected and non-protected forests and semi-natural grasslands (Appendix S3, Section S3.1), through the annual budget for general management of protected areas and subsidies (Jordbruksverket 2021; Skogsstyrelsen 2021a, b). However, agricultural policies are revised every seven years, so long-term funding for grassland management through AES cannot be guaranteed. Personnel at forest companies stated that the companies mostly fund prescribed burning themselves in order to fulfil the criteria for FSC/PEFC-certified forest.

Controls of whether planned disturbances occur or not are only performed in few protected and non-protected forests and grasslands. In protected areas, monitoring of effects of disturbances occurs more extensively (Haglund 2010; Haglund and Vik 2010; Kellner 2012) (Appendix S1, Sections S3.2 and S3.3).

##### Spatial continuity of disturbances

For forests, there are regional strategies specifying which protected areas are to be burned in the future, based on the location of previous fires and regional occurrence patterns of pyrophilous species, with the aim of ensuring continuous supply of burned areas within landscapes (Lindhagen 2009; Berglund 2012). In addition, forest owners with FSC- or PEFC-certified forests are required to establish a landscape plan of their forest holdings, detailing areas appropriate for prescribed fire (Forest Stewardship Council 2020).

For semi-natural grasslands, green infrastructure plans developed by CABs identify landscapes with high total area of semi-natural grasslands and high connectivity between grasslands, and also where connectivity can or should be improved through management activities (Alsén and Kruys 2019; Berlin and Niss 2019; Länsstyrelsen Örebro län 2019).

It is unclear to what extent the strategies detailed above are followed when disturbances are implemented (Appendix S3, Section S3.4). Personnel at both CABs and forest companies state that they try to follow established strategies, but that this is not always possible. Green infrastructure plans have been created only recently. Therefore, it is too early to evaluate how they are used, but there are no subsidies promoting following these plans. Several strategies do not encompass the entire landscape, but only consider the forests or grasslands owned by the organisation responsible for establishing and managing them.

#### Method and intensity of disturbances and post-disturbance treatment

The area burned with both natural and prescribed fires is generally small (mean fire area 3.5 ha or 12 ha, respectively) in both protected and non-protected forest (Ramberg et al. 2018). It is unknown whether there are any differences in fire intensity in protected and non-protected forests. The availability of burned and dead wood is generally higher after fires in protected forests compared to managed forests, as fires in protected forests generally occur in mature forests while a majority of fires in managed forests (including those certified by FSC or PEFC) occur on clear-cuts (Ramberg et al. 2018). Salvage logging does not occur in protected forests, while it sometimes occurs in non-protected forests. For more information on characteristics of disturbances in forests, see Appendix S3, Section S3.5.

Personnel at CABs state that they experience few systematic differences between protected and non-protected semi-natural grasslands regarding management type, intensity, or timing, but that it sometimes is difficult to find grazing animals for protected grasslands. The regulations of management for grasslands receiving AES payments state that management must occur every year and that the remaining vegetation cannot be too tall at the end of the growing season. In addition, the regulations are quite specific, which results in uniform management methods and intensities across all grasslands receiving such payments (Naturvårdsverket 2018). Therefore, in protected areas managed without AES payments it can be easier to tailor management regimes according to the specific requirements of certain species, regarding, e.g. choice of grazing animal species and timing of grazing and mowing.

## Discussion

### Benefits and risks with land sparing and land sharing strategies

We assessed if conservation of disturbance-dependent species is best achieved through setting aside protected areas or by integrating conservation on production land. The literature review revealed higher conservation benefits within protected areas than in production land, but there are many possible factors that may explain this pattern.

There were only rarely differences in effects of fire in protected vs. non-protected forests. This was surprising, since we expected fire in protected areas to generate more high-quality habitat for fire-associated species, especially since many prescribed burnings in managed forest are performed on clear-cuts. Our results could be explained by the fact that to be included a study had to compare the effects of burning in protected and non-protected forests, and only five studies fulfilled this criteria. Previous studies not performed specifically in protected areas have revealed that while disturbance-dependent species can benefit from burning of clear-cuts, benefits are likely to be more pronounced after burning of mature forests as this results in higher amounts of burned and dead wood (Ranius et al. 2014; Heikkala et al. 2017). Thus, further studies are needed in order to evaluate to what extent burnings of production forest and on clear-cuts can substitute burning of protected, mature forests.

For semi-natural grasslands, the studies revealed higher estimates of biodiversity in protected areas. This could be because protection of grasslands leads to management that benefits a larger number of species, but another plausible explanation is that areas selected for protection have higher initial biodiversity (Myers et al. 2000). The latter was a likely explanation for almost two-thirds of reviewed tests, since the non-protected grasslands that were used as contrasts were recently restored grasslands on former agricultural land while the protected grasslands had longer continuity. However, such a comparison can still be relevant, as the contrast can inform about if management of restored grasslands of poorer quality could result in the same conservation values as previously protected grasslands. Still, three studies that evaluated long-term effects in protected and non-protected grasslands with similar history indeed found higher species richness of grassland species in protected areas (de Snoo et al. 2012; Wesche et al. 2012; Krause et al. 2015). Thus, there is at least some evidence that there are more benefits of a land sparing strategy to preserve disturbance-dependent species in semi-natural grasslands.

Overall, a majority of included studies were short-term (< 10 years), but about 40% of studies in grasslands examined differences across 10–50 years. Assessments short after disturbances are relevant for pyrophilous species (Heikkala et al. 2017), and to some extent for saproxylic species, but they should be complemented with later assessments, since observed effects might change over time (Heikkala et al. 2016; Fredriksson et al. 2020). The relatively slow colonisation–extinction dynamics of grassland plant communities (Helm et al. 2006; Cousins 2009) highlights the values of long-term assessments, but many insect species can respond rapidly to, e.g. any changes in grassland management or restoration (Öckinger et al. 2018), suggesting that many studies have indeed been conducted at a relevant time scale.

In many studies, the aim was not to assess how protection status as such affects how biodiversity responds to disturbances. This resulted in many of the included studies being based on the assessment of effects in non-matching areas that may or may not have been a result of the protection, e.g. comparisons of protected versus restored grasslands or comparisons of forests with varying amounts of standing wood prior to burning. In addition, not all studies statistically evaluated the difference between protected and non-protected areas, including all studies performed in forests and nine of the 21 grassland studies. Together, this has implications for the strength of the conclusions based on the empirical studies, and emphasise the need for more empirical evidence on how the protection status of an area per se impacts disturbance effects on biodiversity.

#### Temporal continuity of disturbances

Both grazing- and fire-dependent species depend on temporal continuity of disturbances, albeit on different temporal scales. Maintaining temporal continuity of disturbances is more likely if disturbances are planned in the long term, and if there is sufficient funding. We found that long-term planning of disturbances is generally more extensive with land sparing, as management plans of protected areas state that they are to be protected and managed indefinitely. On non-protected land there is a risk that landowners cease management, especially as the willingness to continue conservation actions often depends on the level of funding (Genghini et al. 2002; Eden 2004; Waldén and Lindborg 2018). Our results indicate that more funding may be available in protected areas, both from compensation payments and from the budget for management of protected areas. However, funds available for maintaining disturbances is variable across years in both protected and non-protected areas.

Climate change is expected to affect the extent and intensity of disturbances, both on protected and non-protected land (Littell et al. 2011). For example, climate warming tends to increase the frequency of natural fires in boreal forests (Kilpeläinen et al. 2010), affecting the future need for prescribed burning and fire suppression (de Groot et al. 2013). Climate change can also increase productivity in semi-natural grasslands in some regions (Dellar et al. 2018; Wang et al. 2019), which in turn may increase the need for management to maintain nutrient-poor conditions and prevent litter accumulation. Thus, flexibility of disturbance regimes may become more important, which might be easier to achieve with a land sharing strategy (Table 2).

**Table 2:** Overview of the main advantages of land sparing and land sharing for the preservation of disturbance-dependent species in forests and semi-natural grasslands, based on a literature review and information from Swedish organisations

	Conclusion	Aspect
Forests	Better with land sparing	Long-term planning of prescribed burning
		Monitoring of fire effects
		Quality of disturbance, but modification of e.g. post-fire treatment could lead to similar benefits with land sparing and sharing
	Better with land sharing	Flexibility in modifying how and when prescribed burning occurs
		Funding of prescribed burning
		Spatial extent and spatial continuity of occurrence of fire
		Less competition with conflicting conservation goals
	Similar with both strategies	Benefits to fire-adapted species
		No follow-up if planned prescribed burnings are performed
Semi-natural grasslands	Better with land sparing	Long-term planning of management
		Monitoring of management effects
		Flexibility in prescribing specific management methods or intensities
		Benefits to grassland species
	Better with land sharing	Spatial extent and spatial continuity of managed grasslands
	Similar with both strategies	Funding of management
		Lack of controls of occurrence of planned management
		Quality of management

#### Spatial extent and continuity of disturbances

Spatial continuity is vital to provide habitat networks where populations can persist in the long term. For grassland species such networks might occur at a smaller scale (within a few kilometres), since many grassland plant species have limited dispersal capability, and their persistence depend on high connectivity (Ozinga et al. 2009; Hooftman et al. 2016; Plue and Cousins 2018; Kimberley et al. 2021). In contrast, for species associated with forest fires, such networks may occur at much larger scales (at least tens of kilometres; Ranius et al. 2014).

Landscape-scale planning of disturbances occurs both with land sparing and land sharing, but it is unclear to us to what extent existing landscape plans are followed when disturbances are implemented. However, since protected areas only cover a small proportion of the total land area in Europe (UNEP-WCMC 2021), it is difficult to obtain spatial continuity of disturbances by only considering them, while more opportunities open up if also considering production land. For example, in Sweden only about 9% of all forest and 5% of all grassland area is formally protected (Christensen et al. 2015; Riksskogstaxeringen 2018; Statistiska Centralbyrån 2018, 2020). Furthermore, only a smaller part of all protected forests are suitable for prescribed burning. This is because the small sizes of many protected forest areas make naturally occurring fires unlikely, and they could not be burned due to difficulty in establishing effective buffer zones between the area and the surrounding forest. Thus, adopting a land sharing strategy can contribute to greater spatial continuity and the extent of disturbances compared to only adopting land sparing (Table 2).

#### Quality of disturbances

Burning of protected areas is typically conducted without felling trees and without subsequent salvage logging, while in production forests, most burnings occur after clear-felling, and salvage logging is more common in cases where mature forest is burned (Ramberg et al. 2018). This results in a habitat of higher quality in protected forests as it results in higher amounts of burned and dead wood, which is vital for pyrophilous and saproxylic species (Wikars 2002; Ylisirniö et al. 2012). However, even in production forests it is possible to perform prescribed burning in mature forests without salvage logging, but this makes burning more expensive. On the other hand, as prescribed burning is always expensive, it can be worth striving for high quality when done. Burning of standing forest is also promoted within the FSC certification as forest owners are allowed to count the total area burned at a factor of three when burning standing forest set aside for conservation purposes. The small size of many protected forest areas can make it difficult to favour disturbance-dependent species. For instance, there is a risk that the fire severity, i.e. how much of individual trees are burnt and the total number of trees burned (Keeley 2009), is lower in small areas, even though we are unaware of any studies investigating this.

We found no clear differences in the management method used, or the intensity or timing of management between protected and non-protected grasslands. However, with a land sparing strategy it is generally easier to tailor management regimes based on site-specific requirements in grasslands. In fact, AES have been criticised for resulting in homogenisation of management (Beaufoy et al. 2011; Jakobsson and Lindborg 2015), while requirements differ between grassland species (Sjödin et al. 2008). In addition, some farmers consider subsidies too low to compensate for incurred production losses (Naturvårdsverket 2018), resulting in grassland abandonment or too intense management practices. Relatively small modifications and a higher degree of flexibility in the requirements for AES payments for semi-natural grasslands could reduce the risk of this strategy not benefitting grassland species.

Overall, this implies that while the characteristics of disturbances will have more benefits to disturbance-dependent species with land sparing, modifications of disturbance regimes in non-protected areas can lead to benefits of land sharing as well (Table 2).

#### Conflicts with conservation and production goals

A land sparing strategy to benefit disturbance-dependent species risks competing with opposing conservation goals. In protected areas aimed at preserving late-successional species, prescribed burning is rarely performed (Brotons et al. 2003; Bouget et al. 2014). In contrast, the land use in non-protected areas already benefit early-successional habitats and species. For example, forest clear-cuts, road verges, and extensively managed green spaces can provide habitat suitable for disturbance-dependent species (Öckinger et al. 2009; Rubene et al. 2014; Dániel-Ferreira et al. 2020). Thus, implementation of disturbances under a land sharing strategy can result in smaller changes that does not compete with other conservation goals (Table 2).

Both strategies involve costs for management and lost revenues, but only land sparing generates acquisition costs. Thus, land sharing might be economically favourable. In addition, as individual landowners may hesitate setting aside their land for protection (Kamal et al. 2015; Jokinen et al. 2018), attitudes towards conservation actions only requiring small changes to current production practices might be more positive, especially if these are associated with subsidies.

### Combining land sparing and land sharing

The conclusions collated in Table 2 suggest that disturbance-dependent species are best preserved by combining conservation efforts in protected areas and production land. The low areal extent, but often high quality of disturbed habitats in protected areas can be complemented by production land contributing with disturbed habitat. Hence, protected and non-protected areas can play different roles. Protected areas (i.e. land sparing) could contribute with source populations and conservation of the most demanding species, while land sharing could increase connectivity and the total area of disturbed habitats. In total, this results in disturbances covering a larger total area, and improved spatial and temporal continuity, which in turn leads to functional habitat networks for disturbance-dependent species. It is important to note that our conclusion on the benefits of combining land sparing and sharing does not consider the consequences of combining land sparing and sharing on yields, commodity production, economic profits, ecosystem services, or public expenditures. Several previous studies assessing the benefits of land sparing vs. land sharing for the preservation of biodiversity support the benefits of applying a combined strategy (Butsic and Kuemmerle 2015; Kremen 2015; Law et al. 2017; Butsic et al. 2020; Finch et al. 2020). Despite the fact that an approach that combines land sparing and sharing strategies is if often already the de facto practice, many disturbance-dependent species remain threatened (Eide et al. 2020), which shows that there is a need to increase the current extent of disturbances.

Implementation of disturbance regimes should be made with the dual aims of maximising local habitat quality and ensuring temporal and spatial extent and continuity of disturbances. This requires cooperation between governmental agencies, private companies, and other organisations, as well as with private landowners. This can be achieved by setting common conservation goals, developing general strategies for prioritisation of conservation measures, and coordinating conservation actions across administrative levels and among landowners (Strange et al. 2006; Cooke et al. 2012). This type of cooperation also has the potential of reducing costs and area requirements to meet conservation objectives and improve species representation in conservation efforts (Bladt et al. 2009; Kark et al. 2015).

## Supplementary Information

Below is the link to the electronic supplementary material.

Supplementary file1 (PDF 748 kb)
