# Supplementary material for: Land sharing complements land sparing in the conservation of disturbance-dependent species
Source: Ambio. 2022 Dec 24;52(3):571–84. doi: 10.1007/s13280-022-01820-1 (PMC9849535; doi:10.1007/s13280-022-01820-1)
Supplement: Supplementary file 1 — Supplementary file1 (PDF 748 kb) [file 13280_2022_1820_MOESM1_ESM.pdf]

Ambio

Supplementary Information

This supplementary information has not been peer reviewed.

Title: Land sharing complements land sparing in the conservation of disturbance-dependent species

Table S1. *The search terms used in the literature searches. Searches were performed in Scopus and using Google and Google Scholar, in English and Swedish. For search terms within brackets, separated by vertical bars, separate searches were performed for each of those terms and the rest of search terms in the search string, i.e. [grazing | mowing] AND biodiversity would mean that two separate searches were performed, one using the search string grazing AND biodiversity, the other mowing AND biodiversity.*

**Forest**	**English**	Scopus	((fire* OR burn* OR wildfire*) AND (forest* OR woodland* OR wood*))<br>**AND**<br>(fire-depend* OR fire-adapt* OR pyroph* OR saprox* OR dead-wood* OR deadwood* OR pyriscence OR heat-trigg* OR fire-trigg* OR wood-living OR wood-feeding OR fire-loving OR fire-favoured OR fire-favored OR woodbor* OR wood-bor*)<br>**AND**<br>(protect* OR natura2000 OR "natura 2000" OR "reserve" OR "reserves" OR "national park*" OR set-aside* OR certifi* OR "voluntary conservation" OR "sustainable forest*" OR "conservation forest*" OR ((strateg* OR priorit* OR design* OR plan* OR polic* OR management) AND conservation))
		Google	[protected | reserve | “national park” | natura2000] **AND** forest **AND** fire **AND** "fire dependent" OR "fire adapted" OR pyrophile OR pyrophilous OR saproxylic OR dead-wood
	**Swedish**	Google	skog **AND** brand **AND** brandgynnad OR pyrofil
		Google Scholar	skog **AND** brand **AND** brandgynnad OR pyrofil
**Semi-natural grassland**	**English**	Scopus	(graz* OR mow* OR scyth* OR grassland* OR pasture* OR meadow* OR (harvest* AND (grassland* OR meadow*)) OR (cutting AND (grassland* OR meadow*)))<br>**AND**<br>("grassland species*" OR indicator* OR specialist* OR "target species" OR "focal species*" OR management-depend* OR grassland-depend* OR grazing-depend* OR mowing-depend* OR (management w/5 depend*) OR (grassland* w/5 depend*) OR (grazing w/5 depend*) OR (mowing w/5 depend*) OR (disturbance* w/5 depend*))<br>**AND**<br>(protect* OR "reserve" OR "reserves" OR natura2000 OR "natura 2000" OR "national park*" OR ((protect* OR "reserve" OR "reserves" OR natura2000 OR "natura 2000" OR "national park*") AND ((agri-environment* OR AES OR "common agricultural polic*" OR CAP OR ((agricultur* OR farm*) AND (subsidi* OR support))) AND (grassland* OR meadow* OR pasture*))) OR ((strateg* OR priorit* OR design* OR plan* OR polic*OR management) AND conservation))
		Google	[grazing | mowing] **AND** biodiversity **AND** grassland **AND** [protected | reserve | “national park” | natura2000] **AND** agri-environment OR ”common agricultural policy” OR subsidies OR support
	**Swedish**	Google	[reservat | natura2000] **AND** [miljöstöd | miljöersättning] **AND** bete OR slåtter
		Google Scholar	[reservat | natura2000] **AND** [miljöstöd | miljöersättning] **AND** bete OR slåtter

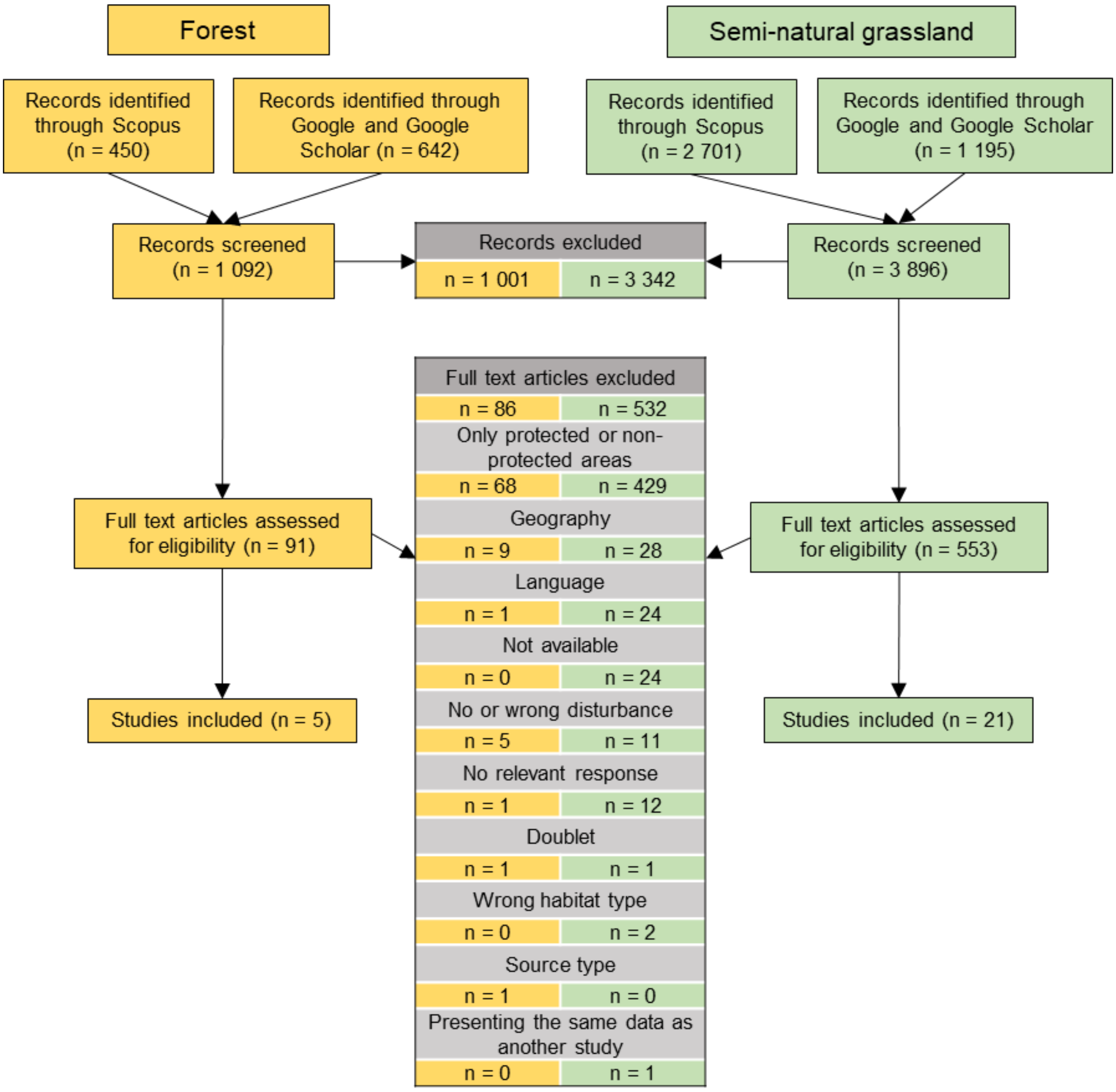

Figure S1. *Overview of review process, with the number of studies retained and excluded at each step.*

Table S2*. List of countries with a majority of their area located in the temperate climate zone of the Northern Hemisphere and thus eligible for inclusion. Definition of the temperate climatic zone was based on Beck et al. (2018), which classifies climatic zones based on temperature and precipitation. The following climatic classifications can be considered as representatives for the temperate zone: oceanic climate (Cfb), subpolar oceanic climate (Cfc), humid continental climate with hot summer subtype (Dfa), humid continental climate with warm summer subtype (Dfb) and subpolar climate (Dfc). Note that while the majority of the USA is not within the temperate zone, we included any study performed in the northeastern part of USA.*

Continent	Country
North America	Canada, USA
Europe	Andorra, Austria, Belarus, Belgium, Bosnia and Herzegovina, Bulgaria, Croatia, Czech Republic, Denmark, Estonia, Finland, France, Germany, Hungary, Ireland, Kosovo, Latvia, Liechtenstein, Lithuania, Luxembourg, Moldova, Montenegro, Netherlands, North Macedonia, Norway, Poland, Romania, Serbia, Slovakia, Slovenia, Sweden, Switzerland, Ukraine, United Kingdom
Asia	Armenia, Georgia, Japan, Russia, Taiwan

Appendix S1. Compilation of interview questions

This appendix compiles the questions that were sent to personnel from governmental agencies, companies and other organizations responsible for maintaining disturbances in Sweden. All questions were not sent to all individuals, rather they were tailored to the expertise of the contacted individual. The questions have been translated from Swedish.

S1.1 General questions

- How is the preservation of disturbance-dependent habitats and species prioritized? For example, which factors determine whether a specific area will be protected or remain non-protected and managed using subsidies?
- How variable is the funding of disturbances in protected and non-protected forests and grasslands? For example, how large are budget variations between years, and does this differ between protected and non-protected areas.

S1.2 Questions related to forests

- Is there an official, national database of the occurrence of natural fires and prescribed burning in protected and non-protected forests?
- How do you finance prescribed burning in protected or non-protected forest? Is there a database of how much forest is burned using governmental subsidies?
- Are there any practical differences between prescribed burning in protected and non-protected forests, e.g. regarding the timing of burning, who performs the burning (e.g. forestry companies, private companies specialized in prescribed burning), and the sensitivity to outside factors that may affect whether or not prescribed burning can occur?
- How common is salvage logging after fire in protected and non-protected forests? Does this differ depending on if the fire was natural or prescribed?
- Do you assess the effects and conservation benefits after fire in protected and non-protected forests, e.g. if occurrence of pyrophilous species increases? How is this assessment performed?
- Can “nature conservation agreements” between private forest owners and the Swedish Forestry Agency or county administration boards specify that prescribed burning should occur in forests with this agreement? How long are these agreements generally valid? Are there any controls of “nature conservation agreements”, that checks if planned fires actually occur? What happens if these agreements are not followed?
- Are there any controls of if planned prescribed burnings actually occur in protected and non-protected forests? How often do these occur? What happens if it is revealed that planned prescribed burnings have not occurred?
- Are there any national or regional strategies available regarding the occurrence of fire in protected and non-protected forests across landscapes, to ensure, e.g., high connectivity between burned areas? To what extent is prescribed burning planned across landscapes? To what extent are these strategies and plans actually followed?
- Does the “ecological landscape plans” for your forest holdings consider prescribed burning, by detailing, e.g., where burning should occur across landscapes?

S1.3 Questions related to semi-natural grasslands

- How is management financed in protected and non-protected grasslands?
- Are there any differences in the ease of getting AES payments for management between protected and non-protected grasslands?
- Are there any practical differences between management of protected and non-protected semi-natural grasslands, e.g. regarding grazing animals, grazing intensity, mowing technique, timing and frequency, the ease of acquiring animals to graze or persons to perform mowing?
- How detailed are the management plans for semi-natural grasslands receiving AES-payments? Do they specify, e.g., management method, intensity or timing? How common is it that payment and management for these grasslands is extended past the initial five or one-year period?
- Are there any controls that check if protected and non-protected grasslands have been managed according to their management plan? How often do these occur? What happens if it is revealed that management has not occurred?
- Are there any national or regional strategies available regarding occurrence of managed protected and non-protected grasslands across landscapes, to ensure, e.g., high connectivity between managed grasslands? To what extent are these strategies actually followed?

Appendix S2. Literature database

Table of all included studies, with details on, e.g., organisms and responses examined, disturbance and main conclusions. Some studies consist of the same experiment, but present results for different organisms, responses or study duration. Snapshot studies compared effects using a single time point. The references to the included studies can be found in the reference list at the end of the supplementary information.

ID	Study	Habitat	Organism	Response	Geography	Appropriate comparison?	Study length	Disturbance	Explicit comparison of protection status?	# protected areas	# non-protected areas	Significance test between protected/ non-protected areas?	Conclusion	Comment
1	Hyvärinen et al. (2005)	Forest	Saproxylic beetles	Change in species richness	Finland	N, unmanaged vs. clear-cut with different levels of tree retention (50, 10, 0 m3/ha)	Snapshot	Prescribed burning	N	3	9	N	Burning of protected forest resulted in slight decrease in species richness of saproxylic beetles; species richness decreased in burned areas without tree retention; species richness increased in burned areas with a tree retention level of 10/50 m3/ha	Same experiment as study 2
			Saproxylic beetles (rare)	Change in species richness	Finland	N, unmanaged vs. clear-cut with different levels of tree retention (50, 10, 0 m3/ha)	Snapshot	Prescribed burning	N	3	9	N	Species richness of rare saproxylic species increased in burned protected and non-protected forest (regardless of tree retention), but increased the most in areas with a retention level of 10 m3/ha	Same experiment as study 2
			Saproxylic beetles	Change in abundance	Finland	N, unmanaged vs. clear-cut with different levels of tree retention (50, 10, 0 m3/ha)	Snapshot	Prescribed burning	N	3	9	N	Abundance of saproxylic beetles was unchanged in burned protected areas, and increased in non-protected areas. Highest increase in areas with a tree retention of 10 m3/ha	Same experiment as study 2
			Saproxylic beetles (rare)	Change in abundance	Finland	N, unmanaged vs. clear-cut with different	Snapshot	Prescribed burning	N	3	9	N	Abundance of rare saproxylic beetles was unchanged in burned	Same experiment as study 2

ID	Study	Habitat	Organism	Response	Geography	Appropriate comparison?	Study length	Disturbance	Explicit comparison of protection status?	# protected areas	# non-protected areas	Significance test between protected/ non-protected areas?	Conclusion	Comment
						levels of tree retention (50, 10, 0 m3/ha)							protected areas, and increased in non-protected areas. Highest increase in areas with a tree retention of 0 m3/ha	
			Saproxylic beetles	Species composition	Finland	N, unmanaged vs. clear-cut with different levels of tree retention (50, 10, 0 m3/ha)	Snapshot	Prescribed burning	N	3	9	N	The species assemblage of saproxylic beetles differed between protected and non-protected forests after burning. Before burning, assemblages were similar in protected and non-protected areas	Same experiment as study 2
2	Hyvärinen et al. (2006)	Forest	Saproxylic beetles (rare, red-listed)	Species richness	Finland	N, unmanaged vs. clear-cut with different levels of tree retention (50, 10, 0 m3/ha)	1 year	Prescribed burning	N	3	9	N	Species richness of rare and threatened saproxylic species was similar (or slightly higher) in protected and non-protected areas one year after burning	Same experiment as study 1, but only focusing on results 1 year after burning
		Forest	Saproxylic beetles (rare, red-listed)	Abundance	Finland	N, unmanaged vs. clear-cut with different levels of tree retention (50, 10, 0 m3/ha)	1 year	Prescribed burning	N	3	9	N	abundance of rare and threatened saproxylic beetles was lower in protected areas compared to non-protected areas with higher tree retention (10, 50 m3/ha)	
3	Erikson et al. (2013)	Forest	Dead wood	Volume	Sweden	Y	1-5 years	Prescribed burning	N	2	1	N	Percentage of dead wood volume (that was present in sites before fire) lost during fire was similar in the protected and non-protected areas. The increase in dead wood volume after fire was highest in one of the PAs (+255%), but in the other PA there was no increase. In the non-protected area, the amount of dead wood doubled after burning, but the overall	

ID	Study	Habitat	Organism	Response	Geography	Appropriate comparison?	Study length	Disturbance	Explicit comparison of protection status?	# protected areas	# non-protected areas	Significance test between protected/ non-protected areas?	Conclusion	Comment
													volume of dead wood was lower than in PAs	
4	Hägglund et al. (2015)	Forest	Saproxylic flatbugs	Species richness	Finland / Sweden	N, protected and non-protected areas in different countries	1-2 years	Prescribed burning	N	2	6	N	Total number of flat bug species was higher in non-protected areas	
		Forest	Saproxylic flatbugs	Abundance	Finland / Sweden	N, protected and non-protected areas in different countries	1-2 years	Prescribed burning	N	2	6	N	Total number of flat bugs was higher in non-protected areas	
		Forest	Coarse woody debris	Volume	Finland / Sweden	N, protected and non-protected areas in different countries	1-2 years	Prescribed burning	N	2	6	N	The increase in amount of coarse woody debris was similar in protected and non-protected areas	
5	Bohman (2009)	Forest	Pyrophilous beetles and flat bugs	Occurrence	Sweden	Y	1-4 years	Natural fire, prescribed burning	N	8	1	N	4 species were found in the non-protected site, similar to the number of species found in protected sites	
6	Saarinen et al. (2016).	Semi-natural grassland	Butterflies (Oarisma poweshiek)	Genetic diversity	Canada / USA	Y	Snapshot	Unknown	N	3	4	N	All populations had low genetic diversity and moderately high levels of inbreeding, with no difference between protected and non-protected areas.	
7	Kiehl and Wagner (2006)	Semi-natural grassland	Plants	Total species richness	Germany	N, protected compared to restored agricultural fields	10 years	Grazing or mowing	Y	2	8	N	Total plant species richness was much higher in protected areas	Same experiment as study 8, 9
		Semi-natural grassland	Plants (grassland species)	Total species richness	Germany	N, protected compared to restored agricultural fields	10 years	Grazing or mowing	Y	2	8	N	Total species richness of grassland plant species was much higher in protected areas	Same experiment as study 8, 9
		Semi-natural grassland	Plants (red-listed)	Total species richness	Germany	N, protected compared to restored agricultural fields	10 years	Grazing or mowing	Y	2	8	N	Total species richness of red-listed plant species was much higher in protected areas	Same experiment as study 8, 9

ID	Study	Habitat	Organism	Response	Geography	Appropriate comparison?	Study length	Disturbance	Explicit comparison of protection status?	# protected areas	# non-protected areas	Significance test between protected/ non-protected areas?	Conclusion	Comment
		Semi-natural grassland	Plants (grassland species)	Proportion grassland species	Germany	N, protected compared to restored agricultural fields	10 years	Grazing or mowing	Y	2	8	N	The proportion of grassland plant species (to all species) was higher in protected areas	Same experiment as study 8, 9
		Semi-natural grassland	Plants (red-listed)	Proportion red-listed species	Germany	N, protected compared to restored agricultural fields	10 years	Grazing or mowing	Y	2	8	N	The proportion of red-listed plant species (to all species) was higher in protected areas	Same experiment as study 8, 9
		Semi-natural grassland	Grasshoppers	Species richness	Germany	N, protected compared to restored agricultural fields	10 years	Grazing or mowing	Y	2	8	N	Species richness of grasshoppers were somewhat higher in protected areas	Same experiment as study 8, 9
		Semi-natural grassland	Grasshoppers	Total species richness	Germany	N, protected compared to restored agricultural fields	10 years	Grazing or mowing	Y	2	8	N	Total species richness of grasshoppers was higher in protected areas	Same experiment as study 8, 9
		Semi-natural grassland	Grasshoppers (grassland species)	Total species richness	Germany	N, protected compared to restored agricultural fields	10 years	Grazing or mowing	Y	2	8	N	Total species richness of grassland grasshopper species was higher in protected areas	Same experiment as study 8, 9
		Semi-natural grassland	Grasshoppers (red-listed)	Total species richness	Germany	N, protected compared to restored agricultural fields	10 years	Grazing or mowing	Y	2	8	N	Total species richness of red-listed grasshopper species was higher in protected areas	Same experiment as study 8, 9
		Semi-natural grassland	Grasshoppers	Density	Germany	N, protected compared to restored agricultural fields	10 years	Grazing or mowing	Y	2	8	N	Density of grasshoppers (ind / 100m) was similar between protected and non-protected areas	Same experiment as study 8, 9
		Semi-natural grassland	Grasshoppers (grassland species)	Proportion grassland species	Germany	N, protected compared to restored agricultural fields	10 years	Grazing or mowing	Y	2	8	N	The proportion of grassland grasshopper species (of all species) was somewhat higher in protected areas	Same experiment as study 8, 9
		Semi-natural grassland	Grasshoppers (grassland species)	Proportion grassland species individuals	Germany	N, protected compared to restored agricultural fields	10 years	Grazing or mowing	Y	2	8	N	Proportion of grassland grasshopper individuals (of all individuals) was higher in protected areas	Same experiment as study 8, 9
		Semi-natural grassland	Grasshoppers (red-listed)	Proportion red-listed individuals	Germany	N, protected compared to restored agricultural fields	10 years	Grazing or mowing	Y	2	8	N	Proportion of red-listed grasshopper individuals (of all individuals) was higher in protected areas	Same experiment as study 8, 9

ID	Study	Habitat	Organism	Response	Geography	Appropriate comparison?	Study length	Disturbance	Explicit comparison of protection status?	# protected areas	# non-protected areas	Significance test between protected/ non-protected areas?	Conclusion	Comment
8	Hofmann et al. (2020)	Semi-natural grassland	Plants (grassland species)	Species richness	Germany	N, protected compared to restored agricultural fields	20 years	Grazing or mowing	Y	1	11	Y	Mean number of grassland plant species were significantly higher in the protected area	Same experiment as study 7, 9
		Semi-natural grassland	Plants	Species richness	Germany	N, protected compared to restored agricultural fields	20 years	Grazing or mowing	Y	1	11	Y	Mean number of plant species was slightly higher in the protected area	Same experiment as study 7, 9
		Semi-natural grassland	Plants (non-grassland species)	Species richness	Germany	N, protected compared to restored agricultural fields	20 years	Grazing or mowing	Y	1	11	Y	Non-grassland species richness (e.g. species of productive grasslands) was at least some times higher in non-protected areas, but for some non-protected areas and the protected area this was very low	Same experiment as study 7, 9
		Semi-natural grassland	Plants (red-listed)	Species richness	Germany	N, protected compared to restored agricultural fields	20 years	Grazing or mowing	Y	1	11	Y	Mean number of red-listed plant species in the protected area was similar to some non-protected areas, but not all, i.e. higher than in some non-protected areas	Same experiment as study 7, 9
		Semi-natural grassland	Plants	Species composition	Germany	N, protected compared to restored agricultural fields	20 years	Grazing or mowing	Y	1	11	Y	Plant species composition differed between the protected and non-protected areas, but over 20 years the composition became more similar	Same experiment as study 7, 9
9	Sieren and Fischer (2002)	Semi-natural grassland	Carabid beetles	Abundance	Germany	N, protected compared to restored agricultural fields	5 years	Grazing or mowing	Y	1	3	Y	Abundance of carabid beetles was higher in non-protected areas	Same experiment as study 7, 8
		Semi-natural grassland	Carabid beetles	Species richness	Germany	N, protected compared to restored agricultural fields	5 years	Grazing or mowing	Y	1	3	Y	Species richness of carabid beetles were higher in non-protected areas	Same experiment as study 7, 8
		Semi-natural grassland	Carabid beetles (grassland species)	Proportion grassland species	Germany	N, protected compared to restored	5 years	Grazing or mowing	Y	1	3	Y	The proportion of grassland carabid species (of all species) was similar in the	Same experiment as study 7, 8

ID	Study	Habitat	Organism	Response	Geography	Appropriate comparison?	Study length	Disturbance	Explicit comparison of protection status?	# protected areas	# non-protected areas	Significance test between protected/ non-protected areas?	Conclusion	Comment
						agricultural fields							protected and non-protected areas	
		Semi-natural grassland	Carabid beetles (non-grassland species)	Proportion non-grassland species	Germany	N, protected compared to restored agricultural fields	5 years	Grazing or mowing	Y	1	3	Y	The proportion of non-grassland carabid species was higher in non-protected areas, the last year there was no significant difference	Same experiment as study 7, 8
		Semi-natural grassland	Carabid beetles (red-listed species)	Proportion red-listed species	Germany	N, protected compared to restored agricultural fields	5 years	Grazing or mowing	Y	1	3	Y	The proportion red-listed carabid species was higher in the protected area the first 3 years, the last 2 there was no significant difference	Same experiment as study 7, 8
10	Horak and Safarova (2015)	Semi-natural grassland	Butterflies	Species richness	Czech republic	Y	Snapshot	Mowing	Y	1	2	Y	Species richness of butterflies was similar in the protected and non-protected areas	
		Semi-natural grassland	Butterflies	Species composition	Czech republic	Y	Snapshot	Mowing	Y	1	2	Y	The species composition differed between the protected and non-protected areas	
11	Leng et al. (2011)	Semi-natural grassland	Plants (grassland species)	Species richness	The Netherlands	Y	Snapshot	Mowing	N	6	18	Y	Species richness of target plant species (ditch bank species) was significantly higher in protected areas compared to areas with conventional management and management financed by agri-environmental subsidies	
12	Fischer et al. (2015)	Semi-natural grassland	Butteflies (Danaus plexippus)	Egg density	USA	N, mowing occurred at different times in protected and non-protected areas (early/late)	Snapshot	Mowing	N	1	1	N	Number of Monarch butterfly eggs per 10 host plants (ramets) was generally higher in the protected area	
13	Zmihorski et al. (2016)	Semi-natural grassland	Birds (grassland species)	Alpha diversity	Poland	Y	Snapshot	Grazing or mowing	Y	221	364	Y	Number of AES grassland bird species was significantly higher in protected areas	

ID	Study	Habitat	Organism	Response	Geography	Appropriate comparison?	Study length	Disturbance	Explicit comparison of protection status?	# protected areas	# non-protected areas	Significance test between protected/ non-protected areas?	Conclusion	Comment
		Semi-natural grassland	Birds (grassland species)	Alpha diversity	Poland	Y	Snapshot	Grazing or mowing	Y	221	364	Y	Number of SPA grassland bird species did not significantly differ in protected and non-protected areas	
		Semi-natural grassland	Birds (grassland species)	Beta diversity	Poland	Y	Snapshot	Grazing or mowing	Y	221	364	Y	Beta diversity of AES grassland bird species was higher in non-protected areas	
		Semi-natural grassland	Birds (grassland species)	Beta diversity	Poland	Y	Snapshot	Grazing or mowing	Y	221	364	Y	Beta diversity of SPA grassland bird species was higher in protected areas	
		Semi-natural grassland	Birds	Occurence	Poland	Y	Snapshot	Grazing or mowing	Y	221	364	Y	4 (of 7) of the most common grassland bird species was more strongly associated with protected than non-protected areas	
14	Resch et al. (2021).	Semi-natural grassland	Plants	Species richness	Switzerland	N, protected compared to restored agricultural fields	20 years	Mowing	Y	Unclear	Unclear	Y	Plant species richness was similar in protected and non-protected areas	
		Semi-natural grassland	Arthropods	Species richness	Switzerland	N, protected compared to restored agricultural fields	20 years	Mowing	Y	Unclear	Unclear	Y	Arthropod species richness was similar in protected and non-protected areas	
15	Wesche et al. (2012)	Semi-natural grassland	Plants	Species richness	Germany	Y	50 years	Grazing or mowing	N	Unclear	Unclear	N	Species richness of plants increased in protected grasslands, and decreased in non-protected areas	Same experiment as study 16
16	Krause et al. (2015)	Semi-natural grassland	Plants (grassland species)	Species richness	Germany	Y	50 years	Grazing or mowing	N	Unclear	Unclear	N	Species richness of grassland plant species increased in protected grasslands, and decreased in non-protected areas	Same experiment as study 15
17	Aerts et al. (1996)	Semi-natural grassland	Birds (Geese)	Abundance	Germany / Netherlands	N, differing grazing intensity in protected and non-protected areas	Snapshot	Grazing	N	1	2	N	Abundance of Anser and Greylag geese was highest in a non-protected, intensively managed area, and lower in the extensively managed protected site. However, abundance was higher in the	

ID	Study	Habitat	Organism	Response	Geography	Appropriate comparison?	Study length	Disturbance	Explicit comparison of protection status?	# protected areas	# non-protected areas	Significance test between protected/ non-protected areas?	Conclusion	Comment
													protected area than in another intensively grazed non-protected area	
18	de Snoo et al. (2012)	Semi-natural grassland	Plants	Species richness	The Netherlands	Y	30 years	Grazing	Y	226	177	Y	Plant species richness was significantly higher in protected areas	
		Semi-natural grassland	Plants	Change in species richness	The Netherlands	Y	30 years	Grazing	Y	226	177	Y	Change in species richness was significantly higher in protected areas	
		Semi-natural grassland	Plants	Floristic value	The Netherlands	Y	30 years	Grazing	Y	226	177	Y	Floristic value was significantly higher in protected areas	
		Semi-natural grassland	Plants	Change in floristic value	The Netherlands	Y	30 years	Grazing	Y	226	177	Y	Change in floristic value was significantly higher in protected areas	
19	Alignan et al. (2018)	Semi-natural grassland	Grasshoppers	Species richness	France	N, protected compared to restored agricultural fields	5 years	Grazing	N	3	3	Y	Species richness of grasshoppers was similar between protected and non-protected areas	
		Semi-natural grassland	Grasshoppers	Abundance	France	N, protected compared to restored agricultural fields	5 years	Grazing	N	3	3	Y	Abundance of grasshoppers was similar in protected and non-protected areas	
		Semi-natural grassland	Grasshoppers	Shannon diversity	France	N, protected compared to restored agricultural fields	5 years	Grazing	N	3	3	Y	Shannon diversity of grasshoppers was similar in protected and non-protected areas	
		Semi-natural grassland	Grasshoppers	Shannon evenness	France	N, protected compared to restored agricultural fields	5 years	Grazing	N	3	3	Y	Shannon evenness of grasshoppers was similar in protected and non-protected areas	
		Semi-natural grassland	Grasshoppers	Species composition	France	N, protected compared to restored agricultural fields	5 years	Grazing	N	3	3	Y	Species composition of grasshoppers was similar in protected an non-protected areas	
20	Leng et al. (2011)	Semi-natural grassland	Plants (grassland species)	Species richness	The Netherlands	N, different management intensity in protected and	5 years	Grazing or mowing	Y	17	25	Y	Species richness of grassland indicator species was unchanged in protected and non-	

ID	Study	Habitat	Organism	Response	Geography	Appropriate comparison?	Study length	Disturbance	Explicit comparison of protection status?	# protected areas	# non-protected areas	Significance test between protected/ non-protected areas?	Conclusion	Comment
						non-protected areas							protected areas, but the species richness was higher in protected areas	
		Semi-natural grassland	Plants (grassland species)	Jaccard similarity	The Netherlands	N, different management intensity in protected and non-protected areas	5 years	Grazing or mowing	Y	17	25	Y	Similarity of species richness between sites (of the same protection status; jaccard index) was unchanged in both protected and non-protected areas, but the similarity was lower in protected (which is positive)	
21	Shevchuk (2018)	Semi-natural grassland	Plants	Total species richness	Ukraine	Y	Snapshot	Grazing	N	1	3	N	Species richness of plants was lowest in the protected area, with almost 100 more species found in non-protected areas	
22	Brereton et al. (2008)	Semi-natural grassland	Butterflies (Polyommatus coridon)	Abundance	England	Y	10 years	Grazing	Y	54	28	Y	The trend in abundance of Polyommatus coridon was similar in protected and non-protected sites, but in sites with AES (both protected and non-protected) abundances increased compared to conventional sites	
23	Schneider and Fry (2001)	Semi-natural grassland	Butterflies (Polyommatus coridon)	Total species richness	Sweden	N, protected and non-protected areas far apart, in different types of landscapes	Snapshot	Grazing or mowing	N	6	5	N	Total species richness of butterflies was higher in non-protected areas	
		Semi-natural grassland	Butterflies (Polyommatus coridon)	Total bundance	Sweden	N, protected and non-protected areas far apart, in different types of landscapes	Snapshot	Grazing or mowing	N	6	5	N	Total abundance of butterfly species was higher in protected areas	
24	Coiffait-Gombault et al. (2012)	Semi-natural grassland	Plants	Species richness	France	N, protected compared to restored agricultural fields	2 years	Grazing	N	1	2	Y	Plant species richness was significantly higher in the protected area	

ID	Study	Habitat	Organism	Response	Geography	Appropriate comparison?	Study length	Disturbance	Explicit comparison of protection status?	# protected areas	# non-protected areas	Significance test between protected/ non-protected areas?	Conclusion	Comment
		Semi-natural grassland	Plants	Species composition	France	N, protected compared to restored agricultural fields	2 years	Grazing	N	1	2	Y	Plant species composition differed between the protected and non-protected areas	
25	Isaksson (2003)	Semi-natural grassland	Carabid beetles	Abundance	Sweden	N, protected and non-protected areas far apart	Snapshot	Grazing or mowing	N	1	2	N	Abundance of carabid beetles was higher in the protected area	
		Semi-natural grassland	Carabid beetles	Species richness	Sweden	N, protected and non-protected areas far apart	Snapshot	Grazing or mowing	N	1	2	N	Species richness of carabid beetles was similar in the protected and non-protected areas	
		Semi-natural grassland	Carabid beetles (red-listed species)	Species richness	Sweden	N, protected and non-protected areas far apart	Snapshot	Grazing or mowing	N	1	2	N	Few red-listed carabid beetle species richness were found in the protected and non-protected areas	
		Semi-natural grassland	Carabid beetles	Species composition	Sweden	N, protected and non-protected areas far apart	Snapshot	Grazing or mowing	N	1	2	N	Species composition of carabid beetles differed between the protected and non-protected areas	
26	O hUallacháin et al. (2016)	Semi-natural grassland	Plants	Species richness	Ireland	Y	Snapshot	Grazing or mowing	Y	20	40	Y	Plant species richness was significantly higher in protected areas	
		Semi-natural grassland	Plants	Shannon diversity	Ireland	Y	Snapshot	Grazing or mowing	Y	20	40	Y	Shannon diversity was significantly higher in protected areas	
		Semi-natural grassland	Plants (grassland species)	Species richness	Ireland	Y	Snapshot	Grazing or mowing	Y	20	40	Y	Species richness of grassland indicator species was significantly higher in protected areas	
		Semi-natural grassland	Plants (non-grassland species)	Species cover	Ireland	Y	Snapshot	Grazing or mowing	Y	20	40	Y	Cover of negative plant indicators was lower in protected areas	
		Semi-natural grassland	Plants	Species composition	Ireland	Y	Snapshot	Grazing or mowing	Y	20	40	Y	Species composition differed between protected and non-protected areas	

Appendix S3. Information from authorities, companies and organisations about governance and implementation of disturbances

S3.1 Differences in funding of disturbances with land sparing and land sharing strategies

There are multiple approaches for funding disturbances when adopting a land sparing strategy in both forests and semi-natural grasslands in Sweden. There is an annual budget for the general management of protected areas, set by the Swedish government and distributed by the Swedish Environmental Protection Agency (Swedish EPA) (Nilsson 2005), but EU LIFE programs (LifeTaiga 2021), and the Swedish framework for conservation of threatened species and habitat types can also be used for funding (Artdatabanken 2021). For land sharing strategies, subsidies for funding prescribed burning in non-protected forest certified by the Forest Stewardship Council (FSC) or Programme for the Endorsement of Forest Certification (PEFC) are available through the Swedish Forestry Agency (SFA) (Skogsstyrelsen 2021a, b), but according to personnel at major forestry companies, prescribed burning to fulfill the requirements of certified forests is usually funded by the companies themselves. In addition, agri-environmental scheme (AES) payments can be used to fund grazing and mowing in both protected and non-protected semi-natural grasslands (Jordbruksverket 2021), but according to personnel at the Swedish Board of Agriculture (SBA) there is no prioritisation of the allocation of AES payments based on protection status. However, if the aim or management plan of a protected grassland conflict with these requirements the opposite may be true. In fact, almost 40 % of semi-natural grasslands in the Swedish Natura 2000-network have to use other funding sources than AES payments to finance management, with conflicts between AES requirements and the management plan of protected areas (Naturvårdsverket 2018). Funding management of non-protected grasslands not receiving AES payments is even more difficult, and only about 50 % of this type of grasslands are managed each year (Glimskär et al. 2017).

Regardless of the funding source and protection status, there is variation in the funds available for maintaining disturbances. Personnel at the Swedish EPA and County Administration Boards (CABs) note that the annual budget for general management of protected areas can be highly variable. For example, in 2019, 860 million SEK were budgeted for conservation efforts such as management of protected areas, while in 2021 the budget was more than twice as large (Miljödepartementet 2019, 2020). In contrast, they state that funding through LIFE projects can be more predictable, as the budget is fixed for the project duration, but that the subsidies available for prescribed burning in non-protected forests can also be variable. According to personnel at SBA, the budget for AES payments for management of semi-natural grasslands is large enough that everyone eligible for payments receive them. Farmers receiving AES payments commit for management of semi-natural grasslands during a five-year period, and as long as requirements are met across this period and there is no change in the area of individual grasslands, the yearly payments stay the same across this period. However, the actual payments are not always large enough to cover the actual cost of grazing or mowing in individual grasslands, as the amount payed is based on the average cost of grazing or mowing of one ha grassland (Jordbruksverket 2021). In addition, the EU Common Agricultural Policy, which govern AES is reformed at regular intervals (European Union 2021), making it more difficult to predict future

compensation levels for management of semi-natural grasslands. Personnel at CABs state that because of the variation in available funding it is more difficult to predict how much will be available for maintaining disturbances in forests and semi-natural grasslands each year, or plan disturbances in the long-term. Some years, there is a risk that not all necessary disturbances can be financed, particularly in protected areas. In these cases, CABs prioritise disturbances of areas requiring annual disturbances (e.g. semi-natural grasslands), rather than areas that are less sensitive to the exact timing of disturbances (e.g. prescribed burning in forests).

S3.2 Differences in controls of the occurrence of disturbances with land sparing and land sharing strategies

There are no guidelines of how often protected forests and semi-natural grasslands should be visited to control whether the disturbances detailed in the management plan actually occurs, but some monitoring of protected areas e.g. focus on the total area burned. In addition, management plans for protected areas often specify that the occurrence of disturbances should be checked. However, the exact method and frequency for controls is generally not specified, and there are no official statistics on how common discrepancies are. Personnel at CABs state that protected areas with higher disturbance frequency and more visitors are generally checked more often, and that if disturbances have not occurred, a solution is found to ensure the occurrence of future disturbances. In addition, personnel at CABs also state that if CABs manage disturbances internally, it is more likely that disturbances occur as they should.

There is an annual audit by FSC/PEFC-certified forest owners. According to personnel at FSC, during this audit the certifier gather documentation from individual forest owners to verify that the registered forest area burned fulfill the certification requirement. If this is not the case, and cannot be justified by, e.g., security reasons (e.g. burning has been impossible due to weather conditions), this has to be remedied. If the total burned area is still too small at the next audit, the forest owner can no longer sell certified timber, and they may have to be re-certified. However, there are no official statistics on how common discrepancies are. One county in northern Sweden (Västerbotten) compile a database of all fires occurring in both protected and non-protected areas within the county, which can be used to control whether or not planned forest fires occur.

According to personnel at SBA and CABs, about 5 % of all semi-natural grasslands managed using AES payments are visited each year, to ensure all requirements are fulfilled. Which grasslands are visited is mainly chosen at random. This means that most visited grasslands are non-protected, and that for a large proportion of grasslands receiving AES payments it is never verified whether management actually occurs. The most common discrepancy is that grazing or mowing have not occurred (yet), or that the management intensity is too low. If this is not rectified in time, this can lead to a deduction of payments, and if management has not occurred for years, AES payments can be cancelled entirely. Several studies have assessed the management in semi-natural grasslands, most of which are likely receiving AES payments, and while some reveal that most surveyed grasslands have sufficient management (Glimskär et al. 2017), others reveal that for many of the surveyed grasslands management was insufficient or lacking (Hägglund et al. 2017; Nordberg 2013; Spörndly and Glimskär 2018; Stenström 2019, 2020). Up to 50 % of semi-natural grasslands without AES payments were not managed (Glimskär et al. 2017).

S3.3 Differences in monitoring of the effects of disturbances with land sparing and land sharing strategies

The Swedish EPA have developed general guidelines for monitoring of protected areas, but also specific manuals for monitoring of individual habitat types. Monitoring of protected areas is financed by the budget for the general management of protected areas, and it is generally recommended that this monitoring is repeated across 12 year intervals (Haglund 2010). In protected forests, monitoring focus on remote sensing to estimate the area of different forest types, but in some forest types the occurrence of fire traces such as burned trees is also monitored. In protected areas with fire prescribed in the management plan, monitoring directly following a natural or prescribed fire is recommended to estimate the total burned area and the total amount of burned and dead wood. It is also recommended that monitoring should be repeated at regular intervals, e.g. to investigate the occurrence of pyrophilous species (Haglund 2010; Kellner 2012). In semi-natural grasslands, monitoring focus on several variables such as aerial image analysis of the cover of shrubs and trees, the vegetation height at the end of the growing season (to ensure annual management actually occurs), and the occurrence of grassland plant, bird and insect species (Haglund 2010; Haglund and Vik 2010). The data from the monitoring is maintained by CABs. Every sixth year, the conservation status of threatened habitats and species listed in the EU Habitats Directive, which are often protected in Natura 2000-areas, has to be reported (Naturvårdsverket 2020). Thus, this also contributes to monitoring of disturbance-dependent habitats and species in protected areas.

According to personnel at forestry companies, monitoring after prescribed fire in FSC/PEFC-certified forests mainly focus on the total area burnt rather than any conservation benefits. However, the forestry companies state that their aim is to standardise and improve the monitoring to also focus on factors such as the amount of burned and dead wood and the occurrence of pyrophilous species. There is no formal monitoring specifically of non-protected semi-natural grasslands (with or without AES payments). However, there is a monitoring program surveying the occurrence of bumblebees, butterflies and plans in approx. 700 semi-natural grasslands across Sweden (Sveriges Lantbruksuniversitet 2021). While this monitoring occurs in both protected and non-protected grasslands with and without AES payments, this data can still provide some general conclusions on the effects of disturbances in semi-natural grasslands.

S3.4 Differences in spatial continuity of disturbances with land sparing and land sharing strategies

Neither management plans for protected areas, the requirements for FSC/PEFC-certified forests nor requirements for semi-natural grasslands managed using AES payments consider the occurrence of disturbances on landscape scales (Forest Stewardship Council 2020; Jordbruksverket 2021). Nevertheless, various strategy documents available from the Swedish EPA, SFA, SBA, CABs and forestry companies do emphasise the importance of planning disturbances across landscapes.

National strategies emphasise the importance of identifying landscapes with high conservation values to guide and prioritise conservation efforts such as prescribed burning and protection of areas (Naturvårdsverket 2013; Naturvårdsverket Skogsstyrelsen 2017; Nitare 2014). There are also strategies related specifically to the occurrence of fire, developed by individual CABs. These specify in which protected areas future prescribed burnings should occur, based on the location and fire history of an area, and the occurrence of pyrophilous species. Often, one aim of these strategies is to ensure a continuous supply of recently burned areas within landscapes (e.g. Berglund 2012; Lindhagen 2009). This topic is sometimes also considered in strategies related to green infrastructure (e.g.

Länsstyrelsen Örebro län 2019). Forest owners with FSC/PEFC-certified forests are required to establish a landscape plan of their forest holdings, detailing forest types and age, location of protected areas and areas appropriate for conservation efforts such as prescribed burning. This information can then be used to plan which areas should be burned to fulfill the area requirement (Forest Stewardship Council 2020). Eco-parks are large areas (at least 1 000 ha) owned by forestry companies, within which they combine regular forestry activities with conservation efforts. These parks have a management plan, and in at least some cases these plans specify the location of future prescribed fires within the parks (Sveaskog 2021). It is unclear how well available regional strategies and landscape plans are actually followed. Personnel at both CABs and forestry companies state that they try to follow them when determining in which areas to perform prescribed burning, but that this is not always possible for security reasons or because areas are too small to burn efficiently without risking fire spread. Still, even if these strategies are not always followed exactly, CABs and forestry companies try to ensure that the occurrence of prescribed burning is spread throughout landscapes. However, individual strategies and plans do not encompass the entirety of the landscape, as these only consider the forests owned by the agency or company responsible for establishing them, and not all forest in the landscape.

In their strategy for preserving biodiversity in the agricultural landscape, SBA emphasise the importance of increasing the area of semi-natural grassland in landscapes, creating and improving dispersal pathways between grasslands, and the importance of preserving valuable grasslands (Wallander et al. 2019). Green infrastructure plans, created by all CABs, also focus on semi-natural grasslands in a wider landscape perspective. These plans identify landscapes with high total area of semi-natural grasslands and high connectivity between grasslands, but also identify where connectivity can or should be improved, through appropriate management of road verges, power line corridors or grasslands fragments (Alsén and Kruys 2019; Berlin and Niss 2019; Länsstyrelsen Örebro län 2019). For all these strategies and plans, it is unclear how well they are followed.

S3.5 Differences in the method and intensity of disturbances between land sparing and land sharing strategies in forests

The total area burned during prescribed fires is generally small, regardless of protection status (average size is 12 ha). However, the forest type burned sometimes differ depending on protection status, which will affect the conservation benefits of a fire: in protected areas, mature and unmanaged forests are generally burned, while 67 % of prescribed burning performed by forestry companies occur on clear-cuts (Ramberg et al. 2018). This type of fire will have limited benefits for pyrophilous and saproxylic species, as there will only be limited availability of burned and dead wood after fire, and as new forests will quickly be planted on burned areas (Heikkala et al. 2017; Heikkala et al. 2016; Hyvärinen et al. 2009; Ranius et al. 2014; Toivanen and Kotiaho 2007). According to the Swedish EPA, only a minority of burnings performed by forestry companies can be classified as conservation burning (Wikars 2006). However, burning on clear-cuts can still have positive effects on some pyrophilous vascular plants and ground-dwelling fungi that require high temperatures for reproduction (Nilsson 2005).

Salvage logging consists of logging of trees that are dead or dying as a result of, e.g. a forests fire or storm felling, to decrease economic losses or prevent outbreaks of pest species such as the spruce bark beetle (Ips typographus) (Lindenmayer et al. 2008). Thus, if salvage logging occurs after a natural or prescribed forest fire, this will limit the benefits to pyrophilous and saproxylic species (Cobb et al. 2010; Cobb et al. 2011; Koivula and Spence 2006; Kotliar et al. 2002; Lindenmayer and Noss 2006;

Thorn et al. 2018), but can still have positive effects on organisms benefitting in open forests, like flowering plants and pollinators (Heil and Burkle 2018). According to personnel at CABs, salvage logging never occurs in protected areas after a fire, unless there is a great risk of pest species outbreaks that could threaten the conservation values in a protected area (Naturvårdsverket 2021). The requirements for FSC/PEFC-certified forest does not specify that salvage logging is forbidden after a fire. However, if enough burned or dead wood is left after a fire (at least 15 % of the tree volume remaining after fire), the net burned area can be multiplied by an upward adjustment factor (Forest Stewardship Council 2020; Programme for the Endorsement of Forest Certification 2017), meaning less area has to be burned to fulfill the certification requirements. Thus, this can create an incentive to avoid salvage logging. However, regulations for production forests limits the amount of dead wood allowed per ha after, e.g., storm felling or fire (maximum 5 m3 conifers per ha) (Skogsstyrelsen 2020). According to personnel at forest companies it varies how common salvage logging is after fire in FSC/PEFC-certified forests: while there is generally no salvage logging after fire in voluntary set-asides, logging sometimes occur in non-set-asides. To what extent salvage logging occurs after natural forest fires depends on the protection status and the size of the fire. After a mega-fire, affecting 13 100 ha of mostly production forest in central Sweden in 2014, about 40 % of the burned area was salvage logged (with most of the rest of the area being protected as a result of the fire) (Gustafsson et al. 2019).

According to personnel at CABs and forestry companies, it is generally challenging to perform prescribed burning regardless of protection status, due to the high security requirements. Prescribed burning in both protected and non-protected forests can be performed by CABs or forestry companies, but most often specific companies are hired. There can be some differences in the flexibility in how and when prescribed burning is performed depending on protection status. For example, according to personnel at forestry companies, flexibility can be higher in non-protected forests, as there is no set management plan that needs to be followed. Fire severity is a factor that affects, e.g., how much of individual trees are burnt and the total number of trees burned, and can affect the response of species and ecosystem processes (Keeley 2009). It is unknown if there are any general differences in the severity of prescribed fires in protected and non-protected forests. However, an assessment from northern Sweden revealed that prescribed fires generally have higher conservation benefits compared to natural forest fires (e.g. as a consequence of higher burn severity) (Sundin 2006).

Appendix S4. References

Aerts, B., Esselink, P., Helder, G., 1996. Habitat selection and diet composition of Greylag Geese Anser anser and Barnacle Geese Branta leucopsis during fall and spring staging in relation to management in the tidal marshes of the Dollard. *Zeitschrift für Ökologie und Naturschutz* 5, 65-75.

Alignan, J.-F., Debras, J.-F., Dutoit, T., 2018. Orthoptera prove good indicators of grassland rehabilitation success in the first French Natural Asset Reserve. *Journal for Nature Conservation* 44, 1-11.

Alsén, M., Kruys, N., 2019. *Grön infrastruktur i Uppsala län*. Länsstyrelsen Uppsala, Uppsala, Sweden.

Artdatabanken, 2021. Åtgärdsprogram för hotade arter, https://www.artdatabanken.se/arter-och-natur/naturvard/atgardsprogram/ (Accessed 2021-10-05).

Beck, H.E., Zimmermann, N.E., McVicar, T.R., Vergopolan, N., Berg, A., Wood, E.F., 2018. Present and future Köppen-Geiger climate classification maps at 1-km resolution. *Scientific Data* 5, 180214.

Berglund, M., 2012. *Strategi för naturvårdsbränning i Jämtlands län 2012–2021*. Länsstyrelsen Jämtlands län, Östersund, Sweden.

Berlin, G., Niss, J., 2019. *Handlingsplan för grön infrastruktur - insatsområden för grön infrastruktur 2019-2030, aktuell period 2019-2022*. Länsstyrelsen Skåne, Malmö, Sweden.

Bohman, P., 2009. *Översiktlig inventering av brandberoende insekter i Västernorrland*. Länsstyrelsen i Västernorrlands län, Härnösand, Sweden.

Brereton, T.M., Warren, M.S., Roy, D.B., Stewart, K., 2008. The changing status of the Chalkhill Blue butterfly Polyommatus coridon in the UK: the impacts of conservation policies and environmental factors. *Journal of Insect Conservation* 12, 629-638.

Cobb, T.P., Hannam, K.D., Kishchuk, B.E., Langor, D.W., Quideau, S.A., Spence, J.R., 2010. Wood-feeding beetles and soil nutrient cycling in burned forests: implications of post-fire salvage logging. *Agricultural and Forest Entomology* 12, 9-18.

Cobb, T.P., Morissette, J.L., Jacobs, J.M., Koivula, M.J., Spence, J.R., Langor, D.W., 2011. Effects of postfire salvage logging on deadwood-associated beetles. *Conservation Biology* 25, 94-104.

Coiffait-Gombault, C., Buisson, E., Dutoit, T., 2012. Using a two-phase sowing approach in restoration: sowing foundation species to restore, and subordinate species to evaluate restoration success. *Applied Vegetation Science* 15, 277-289.

de Snoo, G.R., Naus, N., Verhulst, J., van Ruijven, J., Schaffers, A.P., 2012. Long-term changes in plant diversity of grasslands under agricultural and conservation management. *Applied Vegetation Science* 15, 299-306.

Eriksson, A.-M., Olsson, J., Jonsson, B., Toivanen, S., Edman, M., 2013. Effects of restoration fire on dead wood heterogeneity and availability in three Pinus sylvestris forests in Sweden. *Silva Fennica* 47, 1-15.

European Union, 2021. The common agricultural policy at a glance, https://ec.europa.eu/info/food-farming-fisheries/key-policies/common-agricultural-policy/cap-glance_en (Accessed 2021-10-11).

Fischer, S.J., Williams, E.H., Brower, L.P., Palmiotto, P.A., 2015. Enhancing Monarch Butterfly Reproduction by Mowing Fields of Common Milkweed. *The American Midland Naturalist* 173, 229-240.

Forest Stewardship Council, 2020. *The FSC National Forest Stewardship Standard of Sweden*. Forest Stewardship Council Sweden, Bonn, Germany.

Glimskär, A., Berg, Å., Żmihorski, M., Cronvall, E., Eriksson, Å., 2017. *Kvalitetsförändringar i ängs och betesmarker med och utan miljöersättning*. Jordbruksverket, Jönköping, Sweden.

Gustafsson, L., Berglind, M., Granström, A., Grelle, A., Isacsson, G., Kjellander, P., Larsson, S., Lindh, M., Pettersson, L.B., Strengbom, J., Stridh, B., Sävström, T., Thor, G., Wikars, L.-O., Mikusiński, G., 2019. Rapid ecological response and intensified knowledge accumulation following a north European mega-fire. *Scandinavian Journal of Forest Research* 34, 234-253.

Haglund, A., 2010. *Uppföljning av skyddade områden i Sverige. Riktlinjer för uppföljning av friluftsliv, naturtyper och arter på områdesnivå*. Naturvårdsverket, Stockholm, Sweden.

Haglund, A., Vik, P., 2010. *Manual för uppföljning av betesmarker och slåtterängar i skyddade områden*. Naturvårdsverket, Stockholm, Sweden.

Heikkala, O., Martikainen, P., Kouki, J., 2017. Prescribed burning is an effective and quick method to conserve rare pyrophilous forest-dwelling flat bugs. *Insect Conservation and Diversity* 10, 32-41.

Heikkala, O., Seibold, S., Koivula, M., Martikainen, P., Müller, J., Thorn, S., Kouki, J., 2016. Retention forestry and prescribed burning result in functionally different saproxylic beetle assemblages than clear-cutting. *Forest Ecology and Management* 359, 51-58.

Heil, L.J., Burkle, L.A., 2018. Recent post-wildfire salvage logging benefits local and landscape floral and bee communities. Forest Ecology and Management 424, 267-275.

Hofmann, S., Conradi, T., Kiehl, K., Albrecht, H., 2020. Effects of different restoration treatments on long-term development of plant diversity and functional trait composition in calcareous grasslands. *Tuexenia* 40, 175-200.

Horak, J., Safarova, L., 2015. Effect of reintroduced manual mowing on biodiversity in abandoned fen meadows. *Biologia* 70, 113-120.

Hyvärinen, E., Kouki, J., Martikainen, P., 2006. Fire and green-tree retention in conservation of red-listed and rare deadwood-dependent beetles in Finnish boreal forests. *Conservation Biology* 20, 1711-1719.

Hyvärinen, E., Kouki, J., Martikainen, P., 2009. Prescribed fires and retention trees help to conserve beetle diversity in managed boreal forests despite their transient negative effects on some beetle groups. *Insect Conservation and Diversity* 2, 93-105.

Hyvärinen, E., Kouki, J., Martikainen, P., Lappalainen, H., 2005. Short-term effects of controlled burning and green-tree retention on beetle (Coleoptera) assemblages in managed boreal forests. *Forest Ecology and Management* 212, 315-332.

Hägglund, R., Adler, S., Gardfjell, H., Christensen, P., Cronvall, E., 2017. *Uppföljning av kvalitetsförändringar i ängs och betesmarker via NILS. Tillstånds och förändringsskattningar baserade på data insamlade 2006-2015*. Sveriges Lantbruksuniversitet, Umeå, Sweden.

Hägglund, R., Hekkala, A.-M., Hjältén, J., Tolvanen, A., 2015. Positive effects of ecological restoration on rare and threatened flat bugs (Heteroptera: Aradidae). *Journal of Insect Conservation* 19, 1089-1099.

Isaksson, D., 2003. *Diversitet hos jordlöpare (Col: Carabidae) i hävdade strandnära gräsmarker*. Sveriges lantbruksuniversitet, Uppsala, Sweden.

Jordbruksverket, 2021. Miljöersättning för betesmarker och slåtterängar 2021, https://jordbruksverket.se/stod/lantbruk-skogsbruk-och-tradgard/jordbruksmark/betesmarker-och-slatterangar/betesmarker-och-slatterangar (Accessed 2021-06-11).

Keeley, J., 2009. Fire intensity, fire severity and burn severity: a brief review and suggested usage. *International Journal of Wildland Fire* 18, 116-126.

Kellner, O., 2012. *Manual för uppföljning av skog i skyddade områden*. Naturvårdsverket, Stockholm, Sweden.

Kiehl, K., Wagner, C., 2006. Effect of hay transfer on long-term establishment of vegetation and grasshoppers on former arable fields. *Restoration Ecology* 14, 157-166.

Koivula, M., Spence, J.R., 2006. Effects of post-fire salvage logging on boreal mixed-wood ground beetle assemblages (Coleoptera, Carabidae). *Forest Ecology and Management* 236, 102-112.

Kotliar, N.B., Hejl, S.J., Hutto, R.L., Saab, V., Melcher, C., Mcfadzen, M., 2002. Effects of fire and post-fire salvage logging on avian communities in conifer-dominated forests of the western United States. *Studies in Avian Biology* 25, 49-64.

Krause, B., Culmsee, H., Wesche, K., Leuschner, C., 2015. Historical and recent fragmentation of temperate floodplain grasslands: do patch size and distance affect the richness of characteristic wet meadow plant species? *Folia Geobotanica* 50, 253-266.

Leng, X., Musters, C.J.M., de Snoo, G.R., 2011a. Effects of mowing date on the opportunities of seed dispersal of ditch bank plant species under different management regimes. *Journal for Nature Conservation* 19, 166-174.

Leng, X., Musters, C.J.M., de Snoo, G.R., 2011b. Spatiotemporal variation of plant diversity on ditch banks under different management regimes. *Basic and Applied Ecology* 12, 38-46.

LifeTaiga, 2021. Taiga, http://www.lifetaiga.se/ (Accessed 2021-06-24).

Lindenmayer, D., Burton, P., Franklin, J., 2008. *Salvage Logging and Its Ecological Consequences*. Island Press, Washington DC, USA.

Lindenmayer, D.B., Noss, R.F., 2006. Salvage logging, ecosystem processes, and biodiversity conservation. *Conservation Biology* 20, 949-958.

Lindhagen, A., 2009. *Regional strategi för naturvårdsbränning i skyddade områden Gävleborgs län*. Länsstyrelsen Gävleborg, Gävle, Sweden.

Länsstyrelsen Örebro län, 2019. *Handlingsplan för grön infrastruktur i Örebro län – kunskapsunderlag och åtgärder*. Länsstyrelsen Örebro län, Örebro, Sweden.

Miljödepartementet, 2019. Regleringsbrev för budgetåret 2019 avseende Naturvårdsverket. Available at https://www.esv.se/statsliggaren/regleringsbrev/?rbid=19444.

Miljödepartementet, 2020. Regleringsbrev för budgetåret 2021 avseende Naturvårdsverket. Available at https://www.esv.se/statsliggaren/regleringsbrev/?rbid=21348.

Naturvårdsverket, 2013. *Förvaltning av skogar och andra trädbärande marker i skyddade områden*. Naturvårdsverket, Stockholm, Sweden.

Naturvårdsverket, 2018. *Jordbrukarstöd och värdefulla gräsmarker – hur fungerar de för arbetet med gynnsam bevarandestatus?* Naturvårdsverket Stockholm, Sweden.

Naturvårdsverket, 2020. *Sveriges arter och naturtyper i EU:s art och habitatdirektiv. Resultat från rapportering 2019 till EU av bevarandestatus 2013-2018*. Naturvårdsverket, Stockholm, Sweden.

Naturvårdsverket, 2021. Hantering av granbarkborrar i skyddade områden, https://www.naturvardsverket.se/Miljoarbete-i-samhallet/Miljoarbete-i-Sverige/Uppdelat-efter-omrade/Naturvard/Skydd-av-natur/Hantering-av-granbarkborrar-i-skyddade-omraden/ (Accessed 2021-06-29).

Naturvårdsverket Skogsstyrelsen, 2017. *Nationell strategi för formellt skydd av skog*. Naturvårdsverket Skogsstyrelsen, Stockholm, Sweden.

Nilsson, M., 2005. *Naturvårdsbränning. Vägledning för brand och bränning i skyddad skog*. Naturvårdsverket, Stockholm, Sweden.

Nitare, J., 2014. *Naturvårdande skötsel av skog och andra trädbärande marker*. Skogsstyrelsen, Jönköping, Sweden.

Nordberg, A., 2013. *Utvärdering av ängs och betesmarksinventeringen och databasen TUVA. Hur används TUVA och hur stort är behovet av ominventering?* Jordbruksverket, Jönköping, Sweden.

Ó hUallacháin, D., Finn, J.A., Keogh, B., Fritch, R., Sheridan, H., 2016. A comparison of grassland vegetation from three agri-environment conservation measures. *Irish Journal of Agricultural and Food Research* 55, 176-191.

Programme for the Endorsement of Forest Certification, 2017. *PEFC Sweden Forest Standard*. PEFC Sweden.

Ramberg, E., Strengbom, J., Granath, G., 2018. Coordination through databases can improve prescribed burning as a conservation tool to promote forest biodiversity. *Ambio* 47, 298-306.

Ranius, T., Bohman, P., Hedgren, O., Wikars, L.-O., Caruso, A., 2014. Metapopulation dynamics of a beetle species confined to burned forest sites in a managed forest region. *Ecography* 37, 797-804.

Resch, M.C., Schütz, M., Buchmann, N., Frey, B., Graf, U., van der Putten, W.H., Zimmermann, S., Risch, A.C., 2021. Evaluating long-term success in grassland restoration: an ecosystem multifunctionality approach. *Ecological Applications* 31, e02271.

Saarinen, E.V., Reilly, P.F., Austin, J.D., 2016. Conservation genetics of an endangered grassland butterfly (Oarisma poweshiek) reveals historically high gene flow despite recent and rapid range loss. *Insect Conservation and Diversity* 9, 517-528.

Schneider, C., Fry, G.L.A., 2001. The influence of landscape grain size on butterfly diversity in grasslands. *Journal of Insect Conservation* 5, 163-171.

Shevchuk, N.Y., 2018. Structural comparative analysis of forest and steppe plant communities in the south of Kryvyi Rih region. *Biosystems Diversity* 26, 316-326.

Sieren, E., Fischer, F.P., 2002. Evaluation of measures for enlargement, renaturation and development of a dry grassland biotope by analysing differences in the carabid fauna (Coleoptera). *Acta Oecologica* 23, 1-12.

Skogsstyrelsen, 2020. *Skogsvårdslagstiftningen. Gällande regler 1 april 2020*. Skogsstyrelsen, Jönköping, Sweden.

Skogsstyrelsen, 2021a. Skogens miljövärden, https://www.skogsstyrelsen.se/aga-skog/stod-och-bidrag/skogens-miljovarden/ (Accessed 2021-06-24).

Skogsstyrelsen, 2021b. Stöd till natur- och kulturmiljövårdsåtgärder i skogen (Nokås), https://www.skogsstyrelsen.se/aga-skog/stod-och-bidrag/nokas/ (Accessed 2021-06-24).

Spörndly, E., Glimskär, A., 2018. *Grazing animals and stocking rates in Swedish semi-natural pastures*. Sveriges Lantbruksuniversitet, Uppsala, Sweden.

Stenström, A., 2019. *Miljöövervakning av slåtterängar 2018*. Länsstyrelsen i Västra Götalands län, Göteborg, Sweden.

Stenström, A., 2020. *Miljöövervakning av slåtterängar 2020*. Länsstyrelsen i Västra Götalands län, Göteborg, Sweden.

Sundin, S., 2006. *Spontana eller anlagda bränder, vilka ger mest naturvårdsnytta? – en studie av Gävleborgs län*. Länsstyrelsen Gävleborg, Gävle, Sweden.

Sveaskog, 2021. Våra ekoparker - kombinerar skogsbruk, naturvård och social rekreation, https://www.sveaskog.se/vart-skogsbruk/vart-naturvardsarbete/vara-ekoparker/ (Accessed 2021-06-28).

Sveriges Lantbruksuniversitet, 2021. Om fjärils- och humleinventeringen, https://www.slu.se/institutioner/skoglig-resurshushallning/miljoanalys/fhin/om/ (Accessed 2021-08-03).

Thorn, S., Bässler, C., Brandl, R., Burton, P.J., Cahall, R., Campbell, J.L., Castro, J., Choi, C.-Y., Cobb, T., Donato, D.C., Durska, E., Fontaine, J.B., Gauthier, S., Hebert, C., Hothorn, T., Hutto, R.L., Lee, E.-J., Leverkus, A.B., Lindenmayer, D.B., Obrist, M.K., Rost, J., Seibold, S., Seidl, R., Thom, D., Waldron, K., Wermelinger, B., Winter, M.-B., Zmihorski, M., Müller, J., 2018. Impacts of salvage logging on biodiversity: a meta-analysis. *Journal of Applied Ecology* 55, 279-289.

Toivanen, T., Kotiaho, J.S., 2007. Burning of logged sites to protect beetles in managed boreal forests. *Conservation Biology* 21, 1562-1572.

Wallander, J., Karlsson, L., Berglund, H., Mebus, F., NIlsson, L., Bruun, M., Johansson, L., 2019. *Plan för odlingslandskapets biologiska mångfald*. Jordbruksverket, Stockholm, Sweden.

Wesche, K., Krause, B., Culmsee, H., Leuschner, C., 2012. Fifty years of change in central European grassland vegetation: large losses in species richness and animal-pollinated plants. *Biological Conservation* 150, 76-85.

Wikars, L.-O., 2006. *Åtgärdsprogram för bevarande av brandinsekter i boreal skog*. Naturvårdsverket, Stockholm, Sweden.

Żmihorski, M., Kotowska, D., Berg, Å., Pärt, T., 2016. Evaluating conservation tools in Polish grasslands: The occurrence of birds in relation to agri-environment schemes and Natura 2000 areas. *Biological Conservation* 194, 150-157.
